# Supplementary figures and images for: Klf5 down-regulation induces vascular senescence through eIF5a depletion and mitochondrial fission
Source: PLoS Biol. 2020 Aug 20;18(8):e3000808. doi: 10.1371/journal.pbio.3000808 (PMC7462304; doi:10.1371/journal.pbio.3000808)

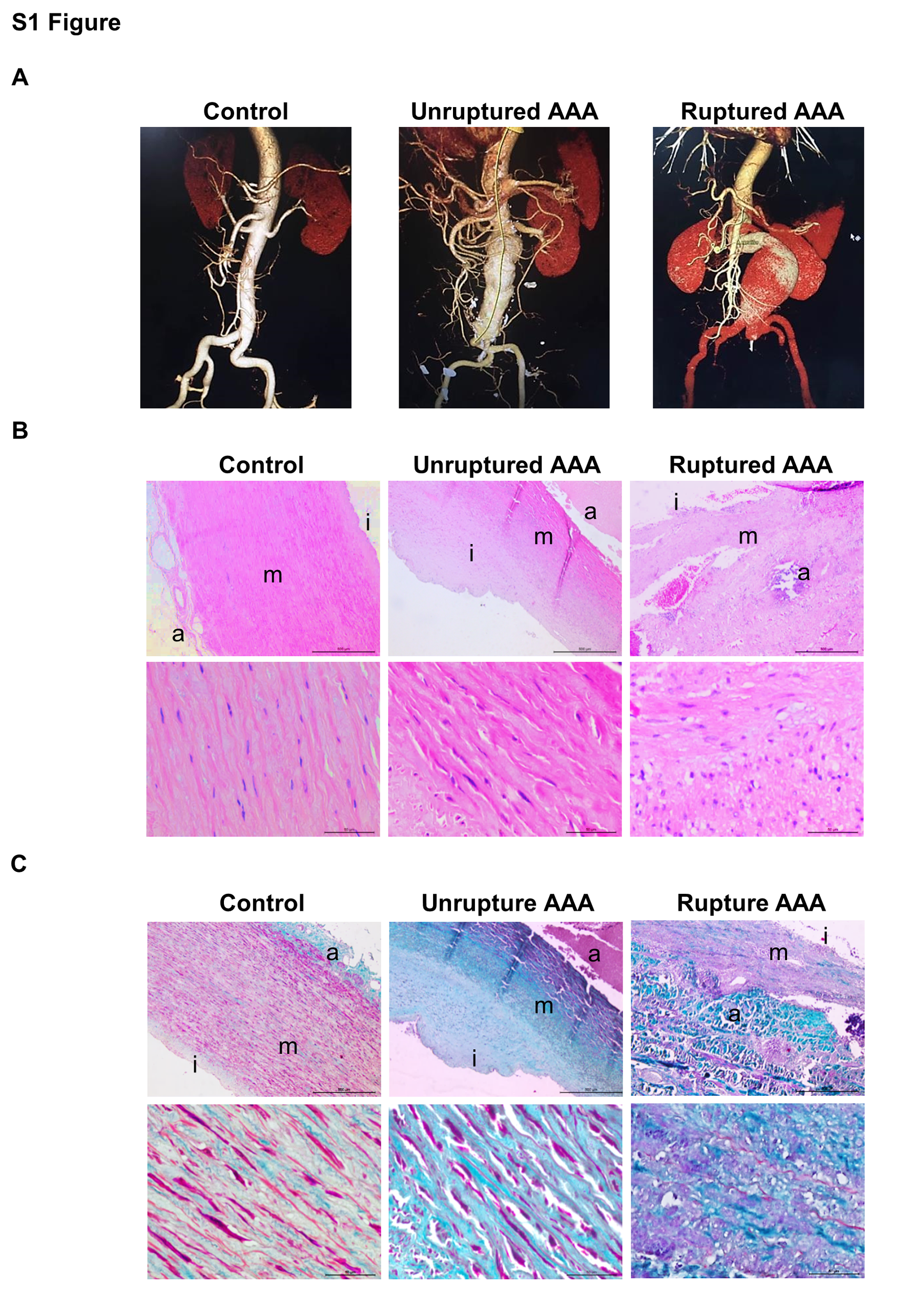

Supplement: S1 Fig — (A) Representative 3D volume-rendered image of abdomen from the normal human abdominal aorta and unruptured and ruptured aneurysms. (B, C) Representative HE-stained and Masson-stained sections from the normal human abdominal aorta and unruptured and ruptured aneurysms. Scale bars = 500 and 50 μm. HE, hematoxylin–eosin. (TIF) [file pbio.3000808.s001.tif]

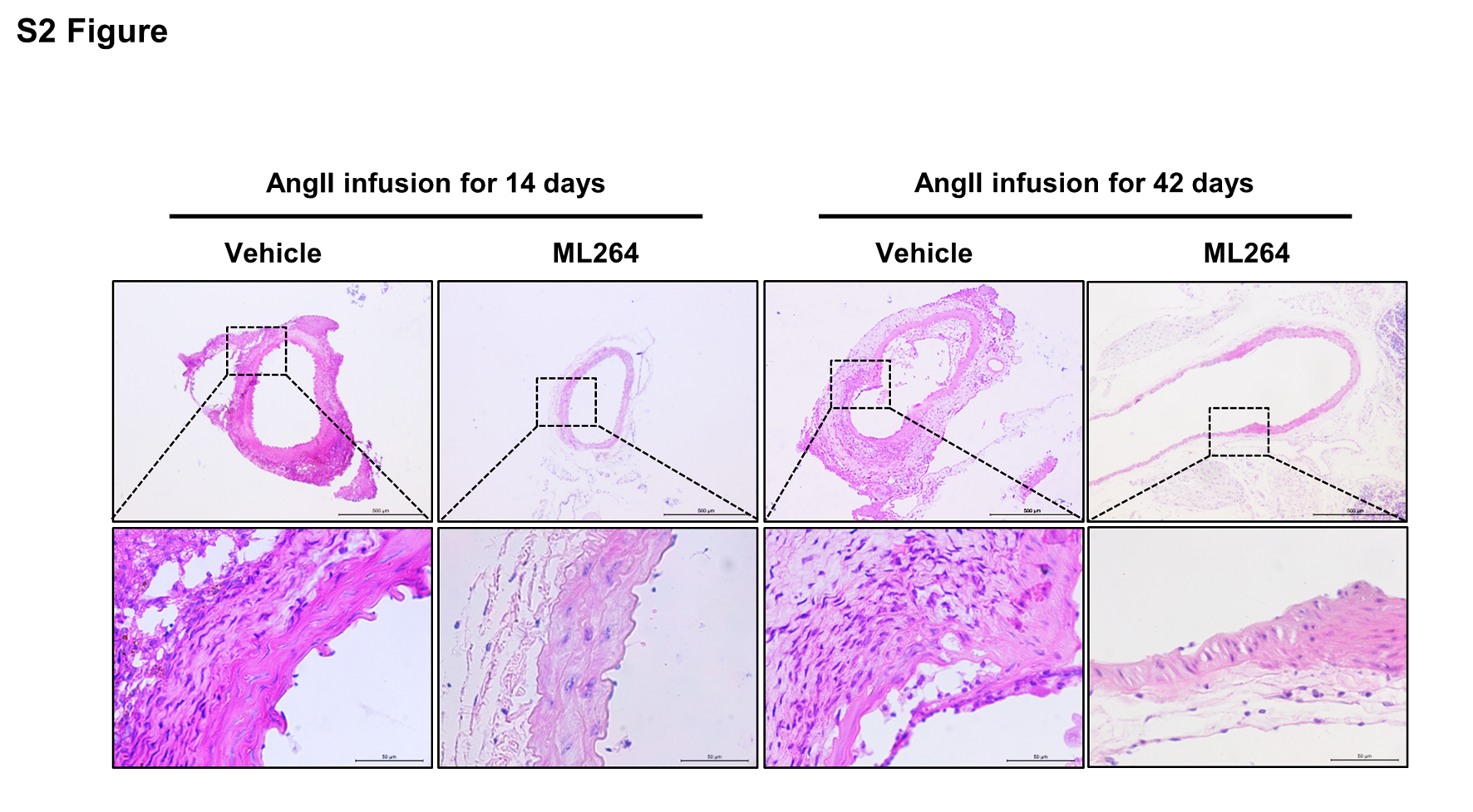

Supplement: S2 Fig — Representative ultrasound imaging of mouse AAA models induced by Ang II infusion for 14 and 42 days in ApoE−/− mice injected intraperitoneally with ML264 every two days for 14 and 42 days. Ang II, angiotensin II; Klf5, Krüppel-like factor 5. (TIF) [file pbio.3000808.s002.tif]

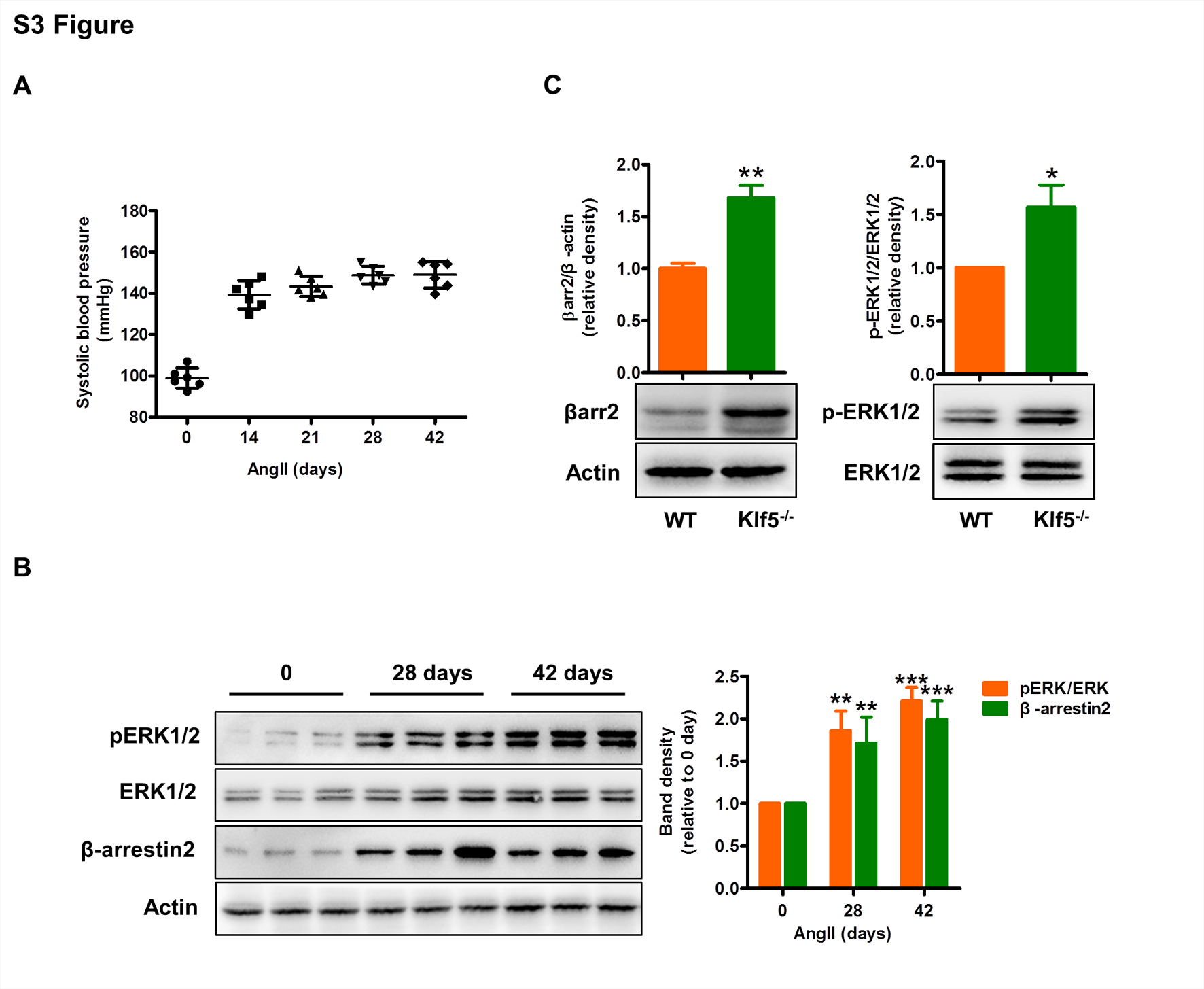

Supplement: S3 Fig — (A) Systolic blood pressure measured by tail cuff method in conscious mice following 0, 14, 21, 28, 42 days of Ang II infusion. (B) The expression of β-arrestin2 and ERK1/2 was analyzed by western blotting in Ang II–injured mouse aortas for 28 and 42 days. β-actin was used as a loading control. **P < 0.01 and ***P < 0.01 versus 0 day. (C) The expression of β-arrestin2 and ERK1/2 was analyzed by western blotting in WT and Klf5−/− VSMCs. *P < 0.05 and **P < 0.01 versus WT. For numerical raw data, please see S1 Data. For raw immunoblots, please see S1 Blots. AAA, abdominal aortic aneurysm; Ang II, angiotensin II; ERK, extracellular signal–regulated kinase; Klf5, Krüppel-like factor 5; VSMC, vascular smooth muscle cell; WT, wild-type. (TIF) [file pbio.3000808.s003.tif]

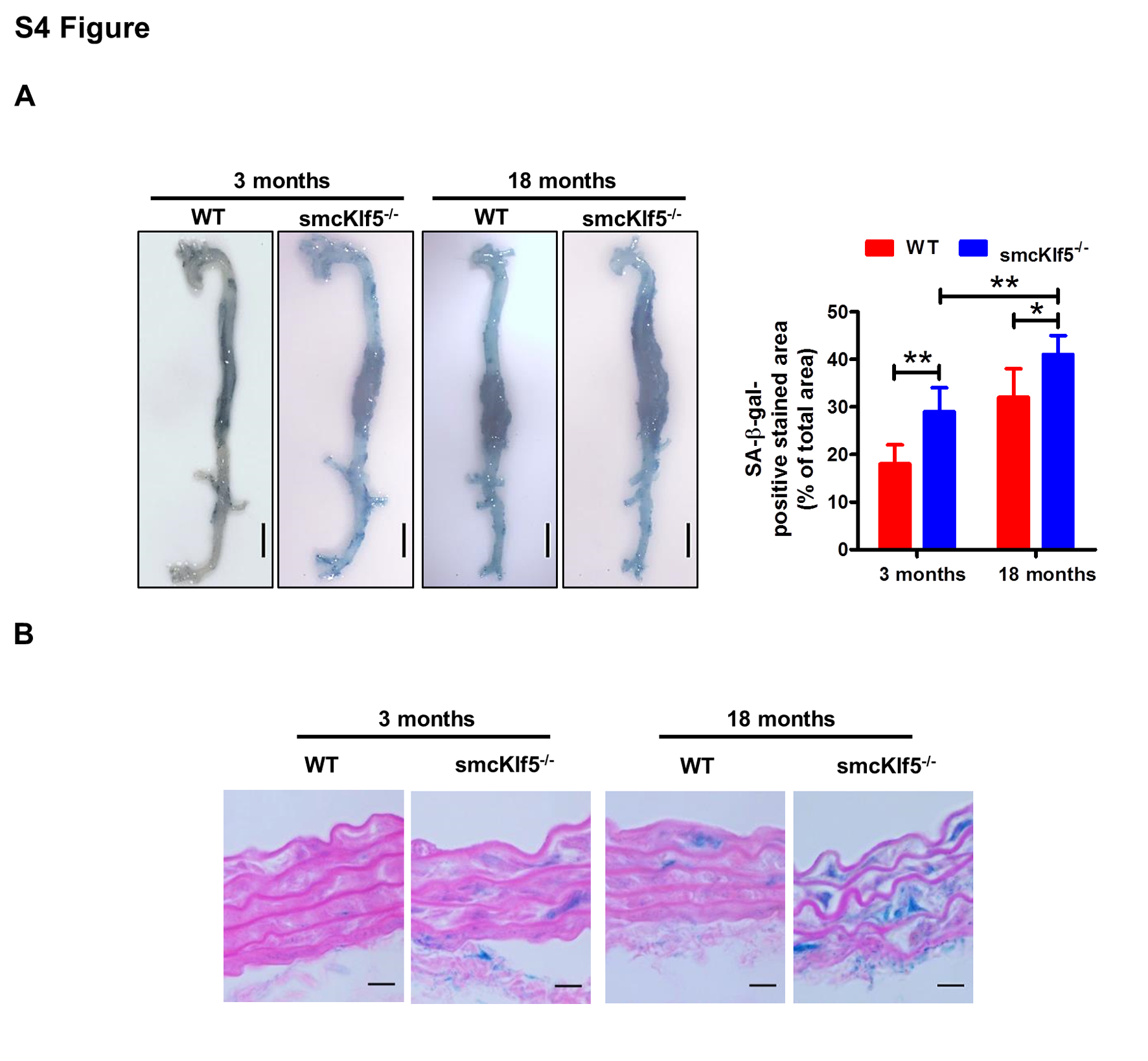

Supplement: S4 Fig — (A) Representative photographs and quantitative analysis of SA-β-gal–stained aortas from WT and smcKlf5−/− mice. Scale bars = 5 mm; n = 5 per group, *P < 0.05 and **P < 0.01 versus WT or young smcKlf5−/− mouse. (B) Representative images of SA-β-gal–stained transverse sections of abdominal aortas from WT and smcKlf5−/− mice. Blue staining indicates SA-β-gal–positive stained cells, and cytoplasm and extracellular matrix were counterstained using HE. Scale bars = 50 μm. For numerical raw data, please see S1 Data. Ang II, angiotensin II; HE, hematoxylin–eosin; SA-β-gal, senescence-associated β-galactosidase; WT, wild-type. (TIF) [file pbio.3000808.s004.tif]

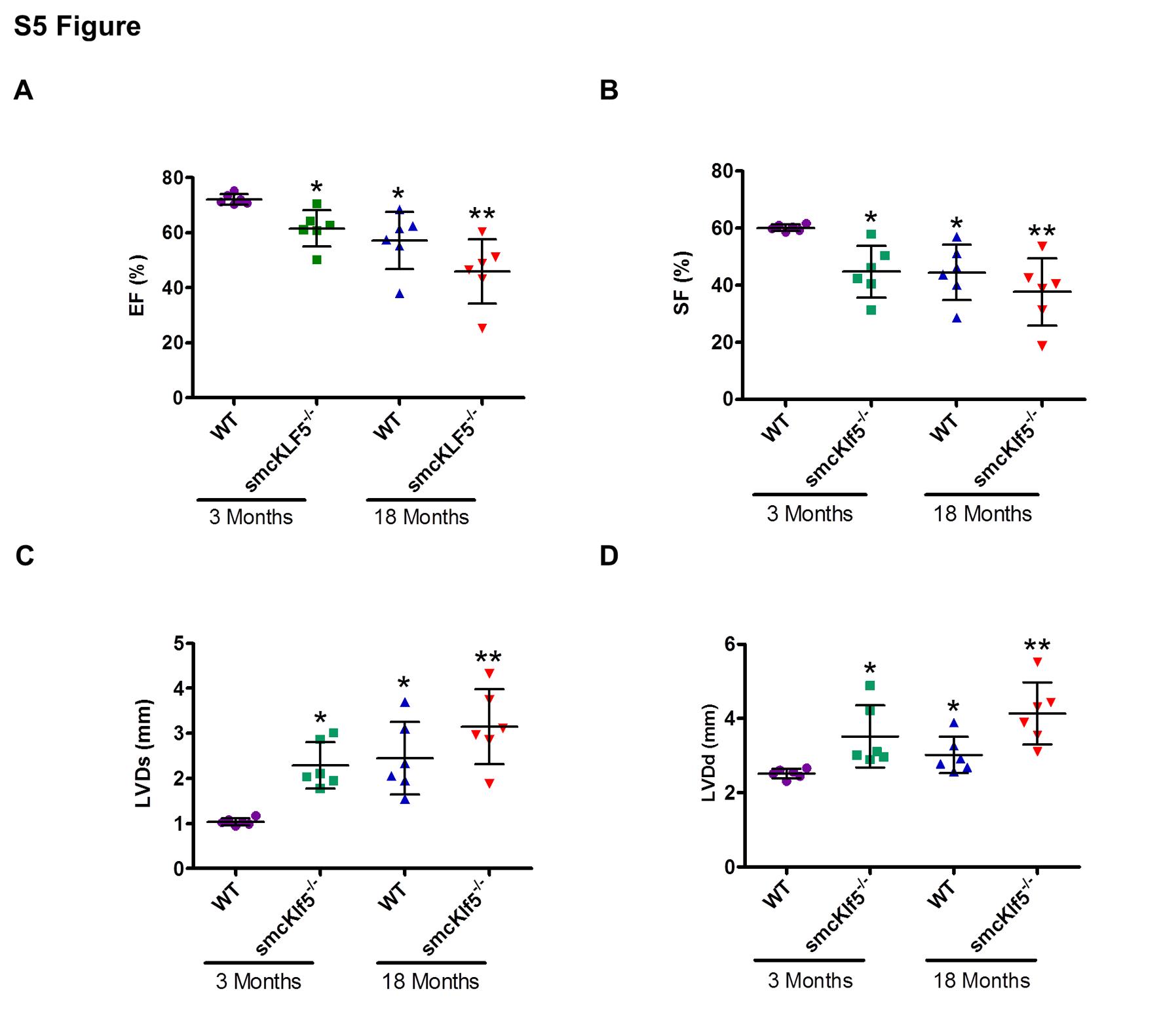

Supplement: S5 Fig — (A) Ejection fraction, (B) shortening fraction, (C) left ventricular dimension at systole, (D) left ventricular dimension at diastole. *P < 0.05, **P < 0.01 versus WT. n = 6 for each group. For numerical raw data, please see S1 Data. WT, wild-type. (TIF) [file pbio.3000808.s005.tif]

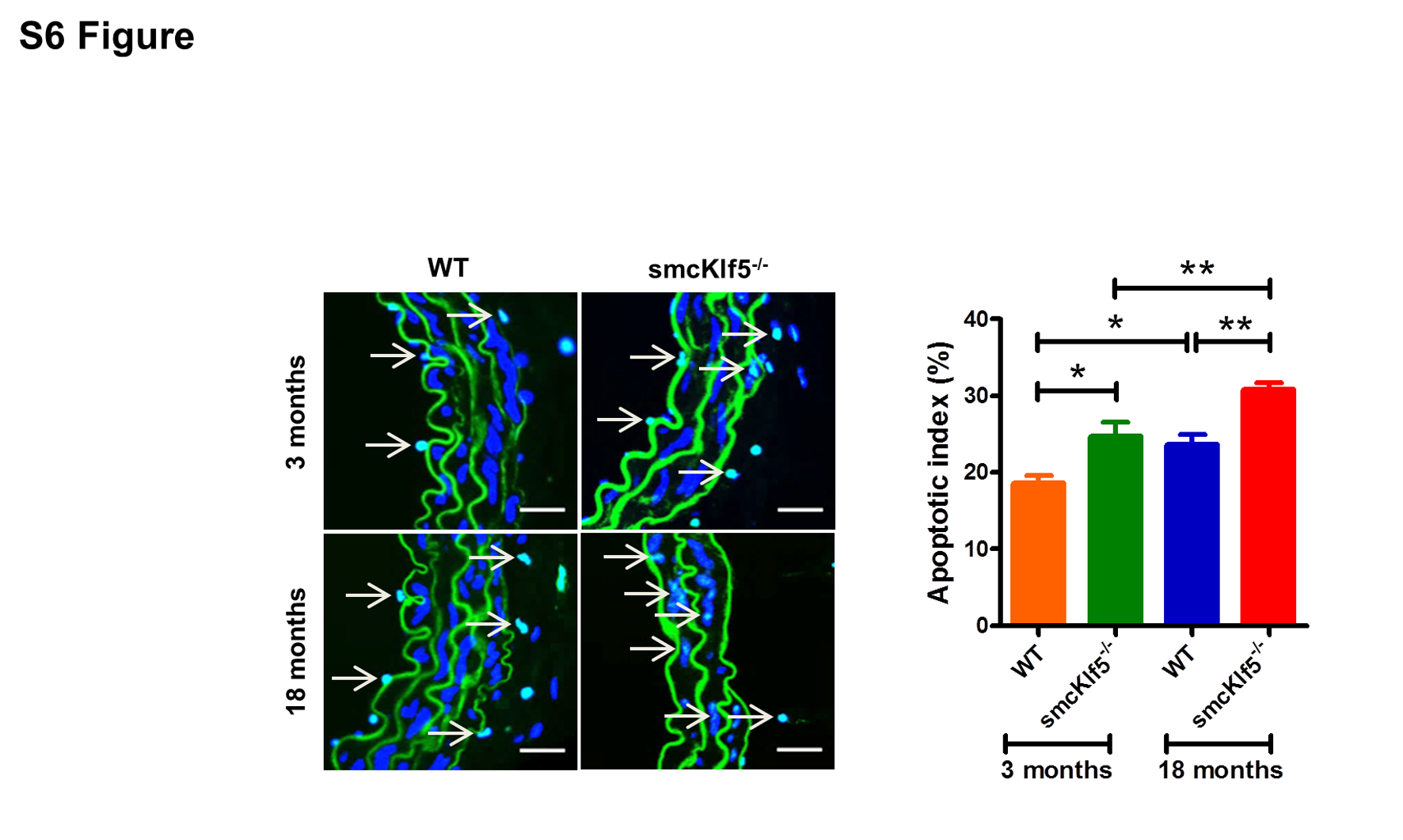

Supplement: S6 Fig — Graphical data represent the percentage of apoptotic cells (green)/the total number of nucleated cells (blue). n = 3 in each group, *P < 0.05 and **P < 0.01 versus WT or young mice. Scale bars = 50 μm. For numerical raw data, please see S1 Data. Ang II, angiotensin II; WT, wild-type. (TIF) [file pbio.3000808.s006.tif]

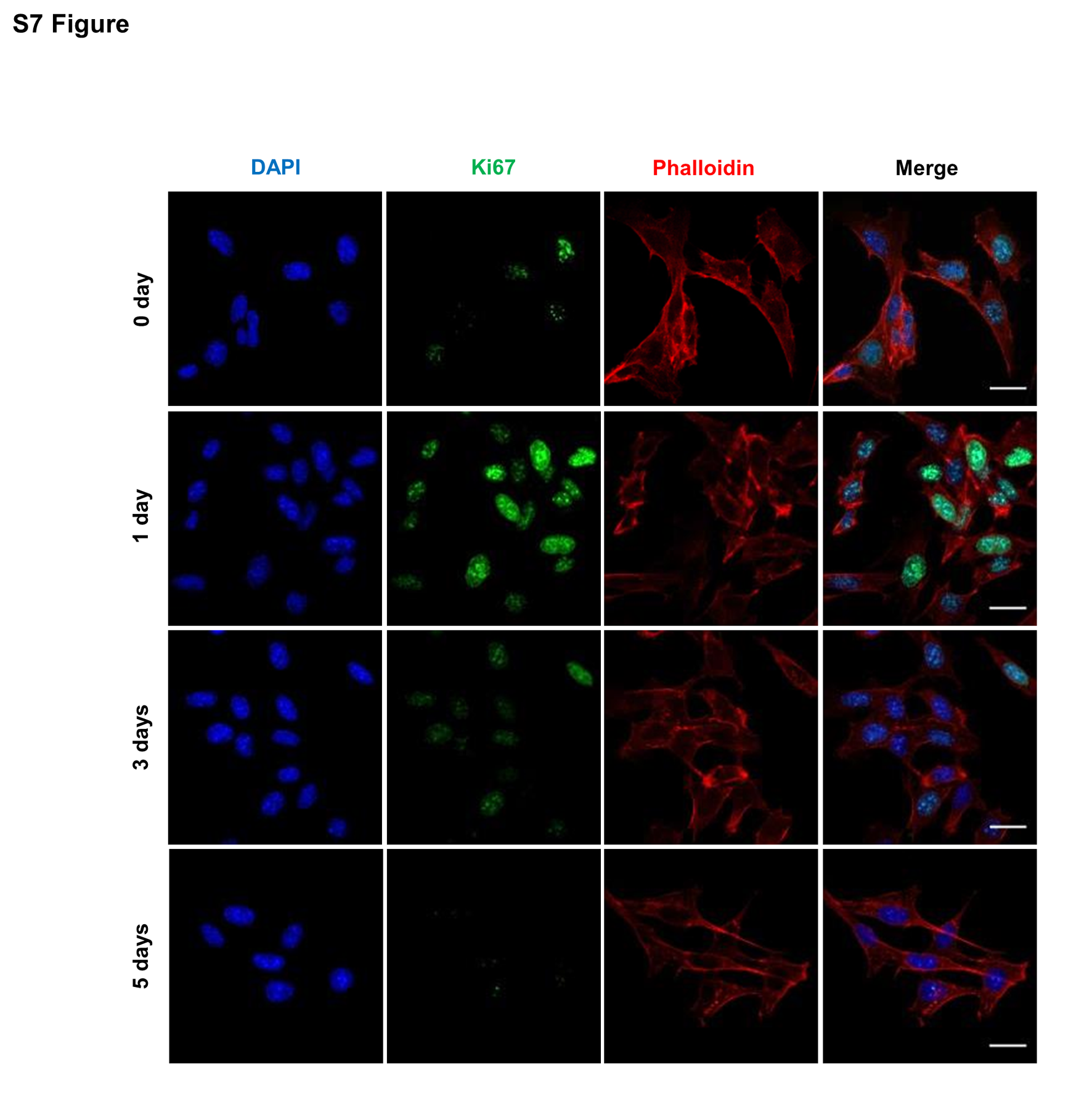

Supplement: S7 Fig — Representative immunofluorescent images of Ki67 (green) and phalloidin (red) staining of VSMCs treated with Ang II. Scale bars = 5 μm. Ang II, angiotensin II; VSMC, vascular smooth muscle cell. (TIF) [file pbio.3000808.s007.tif]

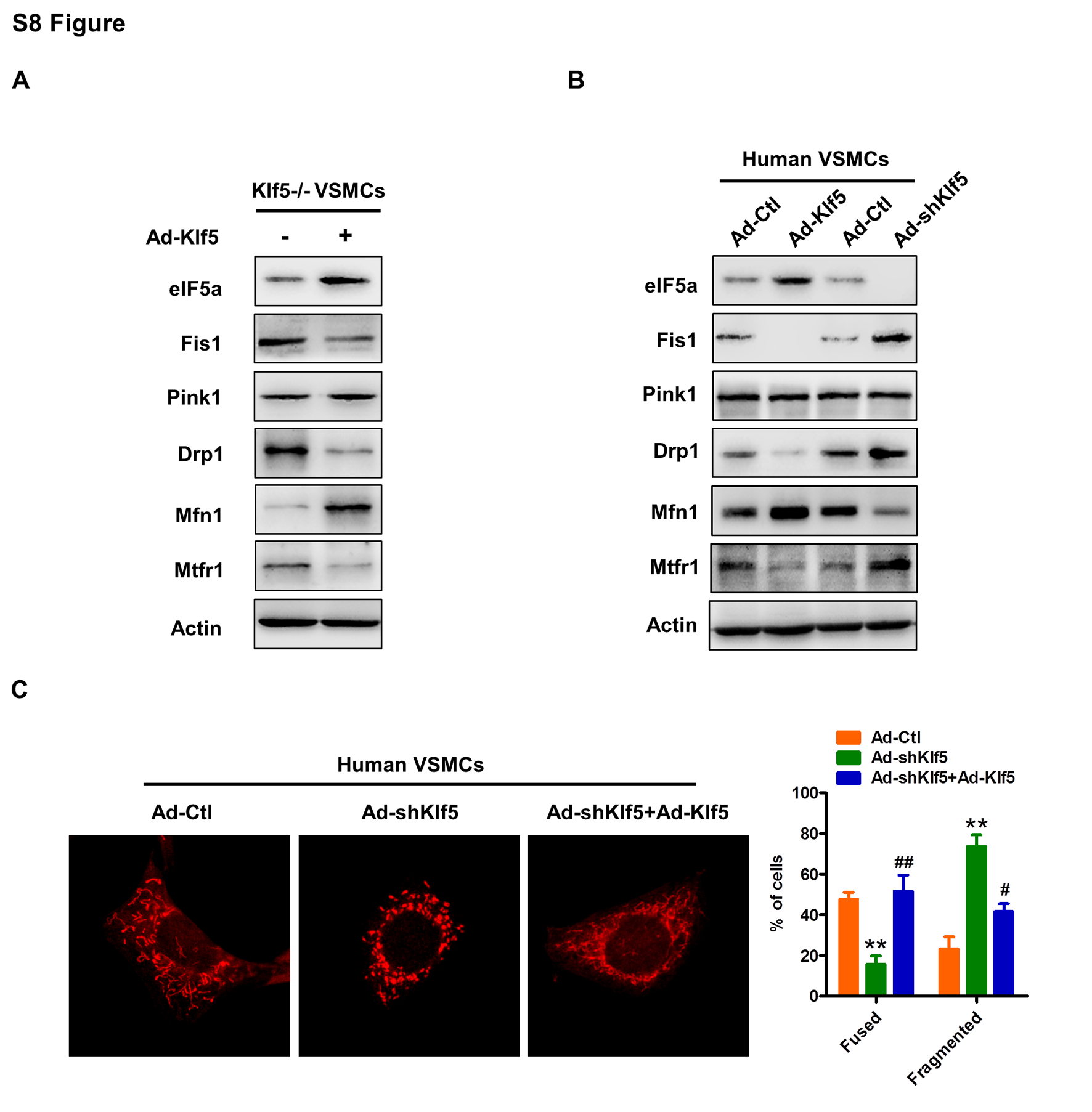

Supplement: S8 Fig — (A) Representative western blot image of eIF5a, Fis1, Pink1, Drp1, Mfn1, and Mtfr1 in Klf5−/− VSMCs infected or not with Ad-Klf5. (B) Representative western blot image of eIF5a, Fis1, Pink1, Drp1, Mfn1, and Mtfr1 in human VSMCs infected with Ad-Klf5 and Ad-Ctl or Ad-shKlf5. (C) MitoTracker Red–stained mitochondria in VSMCs infected with indicated constructs. Right: the percentage of cells containing fused and fragmented mitochondria was quantified from more than 100 cells. Scale bars = 10 μm. Data represent mean ± SEM, **P < 0.01 versus Ad-Ctl; #P < 0.05 and ##P < 0.01 versus Ad-shKlf5. For numerical raw data, please see S1 Data. Ad-Ctl, adenoviruses encoding control; Ad-Klf5, adenoviruses encoding Klf5; Ad-shKlf5, adenoviruses encoding small hairpin Klf5; Drp1, dynamin-related protein 1; eIF5a, eukaryotic translation initiation factor 5a; Fis1, fission mitochondrial 1; Klf5, Krüppel-like factor 5; Mfn1, mitofusin 1; Mtfr1, mitochondrial fission regulator 1; Pink1, PTEN-induced kinase 1; VSMC, vascular smooth muscle cell. (TIF) [file pbio.3000808.s008.tif]

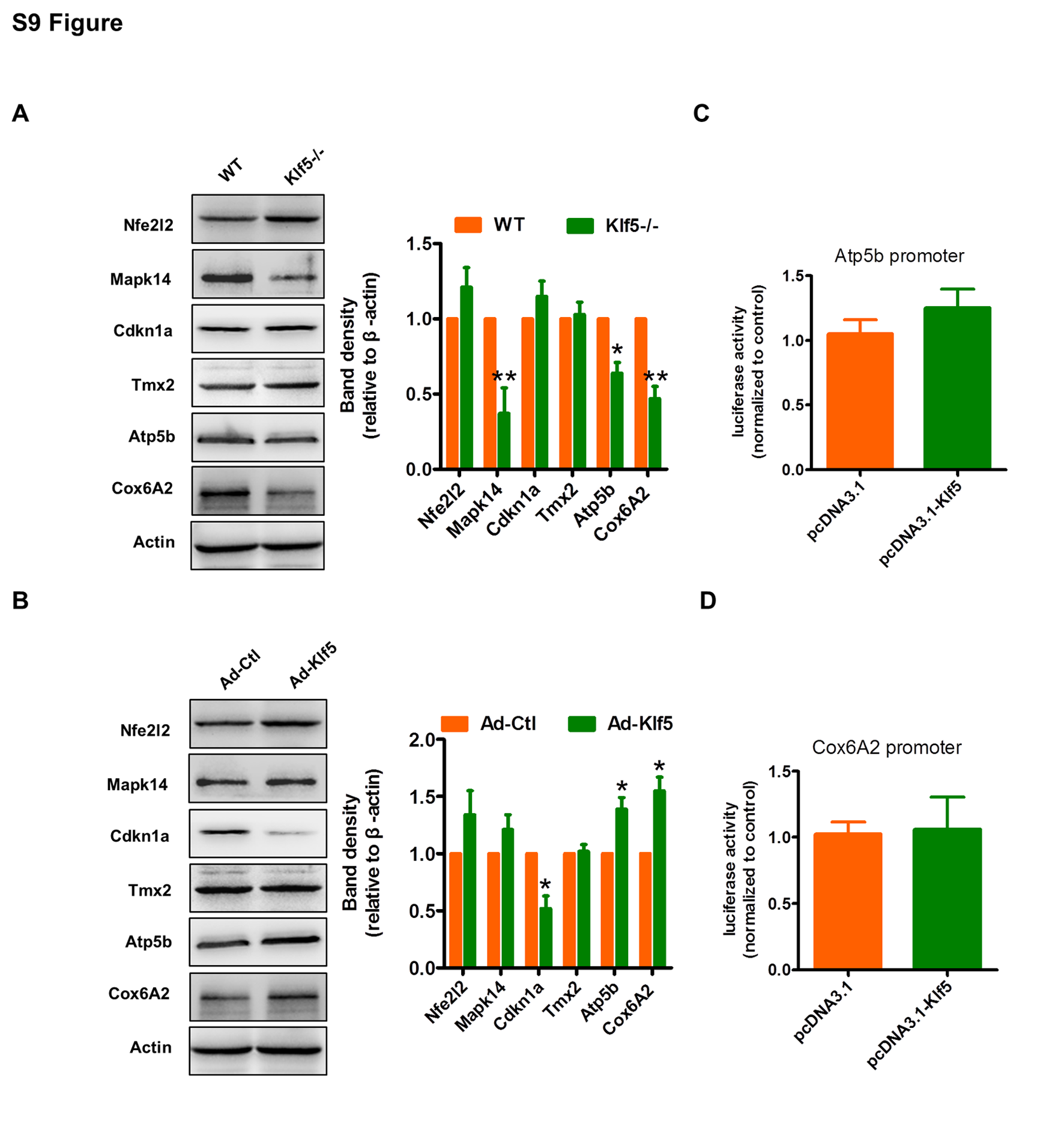

Supplement: S9 Fig — (A) Representative western blot image of Nfe2l2, Mapk14, Cdkn1a, Tmx2, Atp5b, and Cox6a2 in WT and Klf5−/− VSMCs. Right: Band intensities that were measured and normalized to β-actin (n = 3). Data represent the mean ± SD. *P < 0.05 and **P < 0.01 versus WT. (B) Representative western blot image of Nfe2l2, Mapk14, Cdkn1a, Tmx2, Atp5b, and Cox6a2 in Ad-Klf5– and Ad-Ctl–infected mouse VSMCs. Right: Band intensities that were measured and normalized to β-actin (n = 3). *P < 0.05 versus Ad-Ctl. (C,D) Cells (293A) were transfected with the reporter directed by the Atp5b (C) or Cox6a2 (D) promoter, and luciferase activity was measured. Data represent the relative eIF5a or Cox6a2 promoter activity normalized to pRL-TK activity. For numerical raw data, please see S1 Data. Ad-Ctl, adenoviruses encoding control; Ad-Klf5, adenoviruses encoding Klf5; Atp5b, ATP synthase subunit β; Cdkn1a, cyclin dependent kinase inhibitor 1a; Cox6a2, cytochrome c oxidase subunit 6A isoform 2; eIF5a, eukaryotic translation initiation factor 5a; Klf5, Krüppel-like factor 5; Mapk14, mitogen-activated protein kinase 14; Nfe2l2, nuclear factor, erythroid 2 like 2; pRL-TK, thymidine kinase promoter-Renilla luciferase reporter plasmid; Tmx2, thioredoxin-related transmembrane protein 2; VSMC, vascular smooth muscle cell; WT, wild-type. (TIF) [file pbio.3000808.s009.tif]

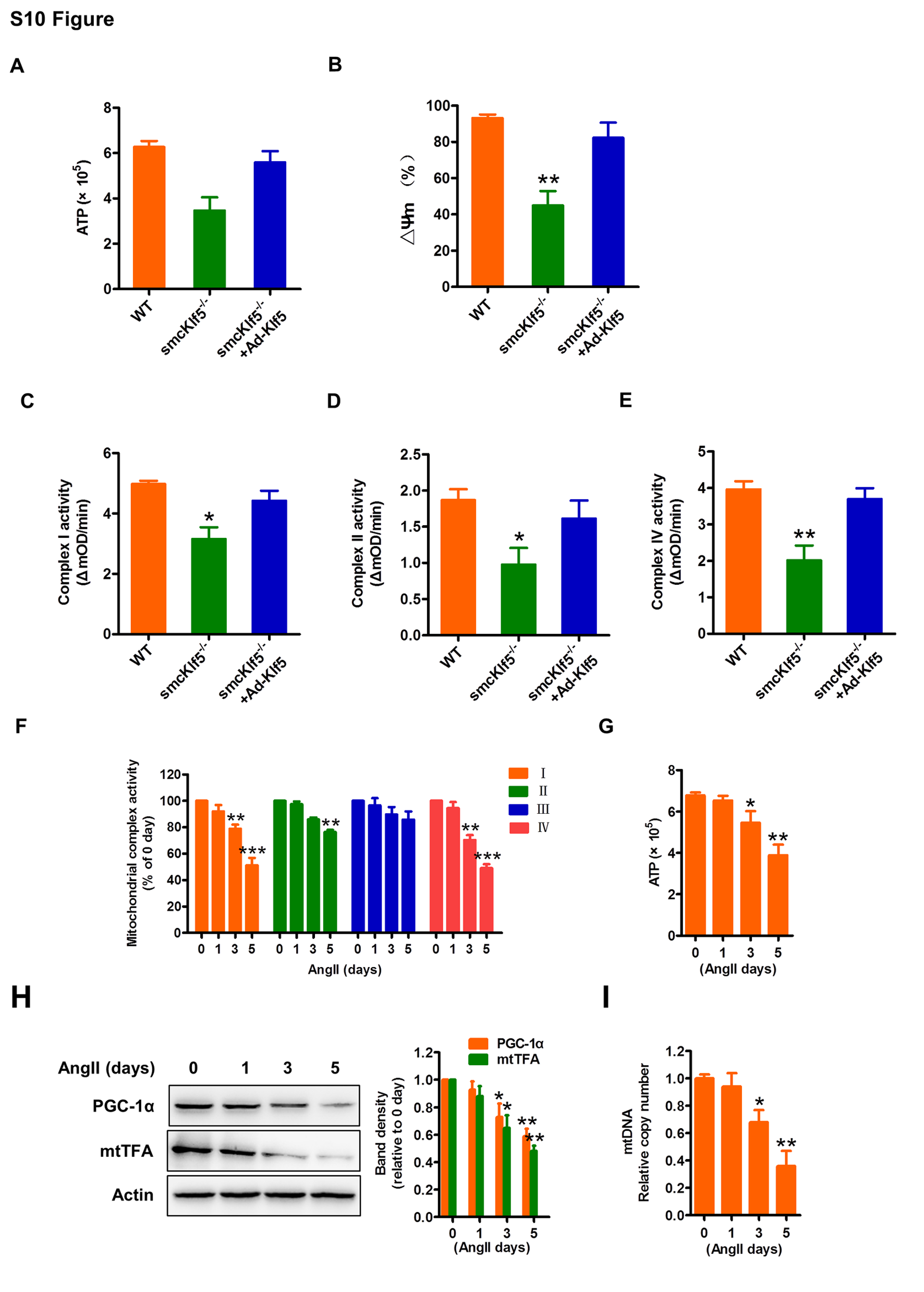

Supplement: S10 Fig — (A-E) Analysis of ATP content (A), relative ΔΨm (B), and activities of complexes I, II, and IV (B-D) in WT and Klf5−/− VSMCs as well as in Ad-Klf5–infected Klf5−/− VSMCs. *P < 0.05 and **P < 0.01 versus WT. (F-I) Mouse VSMCs were stimulated with Ang II (100 nmol/L) for the indicated times, and then the activities of complexes I, II, III, and IV (F), ATP content (G), the expressions of PGC-1α and mtTFA (H), and the copy number of mtDNA (I) were analyzed. *P < 0.05, **P < 0.01, and ***P < 0.001 versus 0 day. For numerical raw data, please see S1 Data. For raw immunoblots, please see S1 Blots. Ad-Klf5, adenoviruses encoding Klf5; Ang II, angiotensin II; Klf5, Krüppel-like factor 5; mtDNA, mitochondrial DNA; mtTFA, mitochondrial transcription factor A; PGC-1α, peroxisome proliferative activated receptor, gamma, coactivator 1 alpha; VSMC, vascular smooth muscle cell; WT, wild-type; ΔΨm, mitochondrial membrane potential. (TIF) [file pbio.3000808.s010.tif]

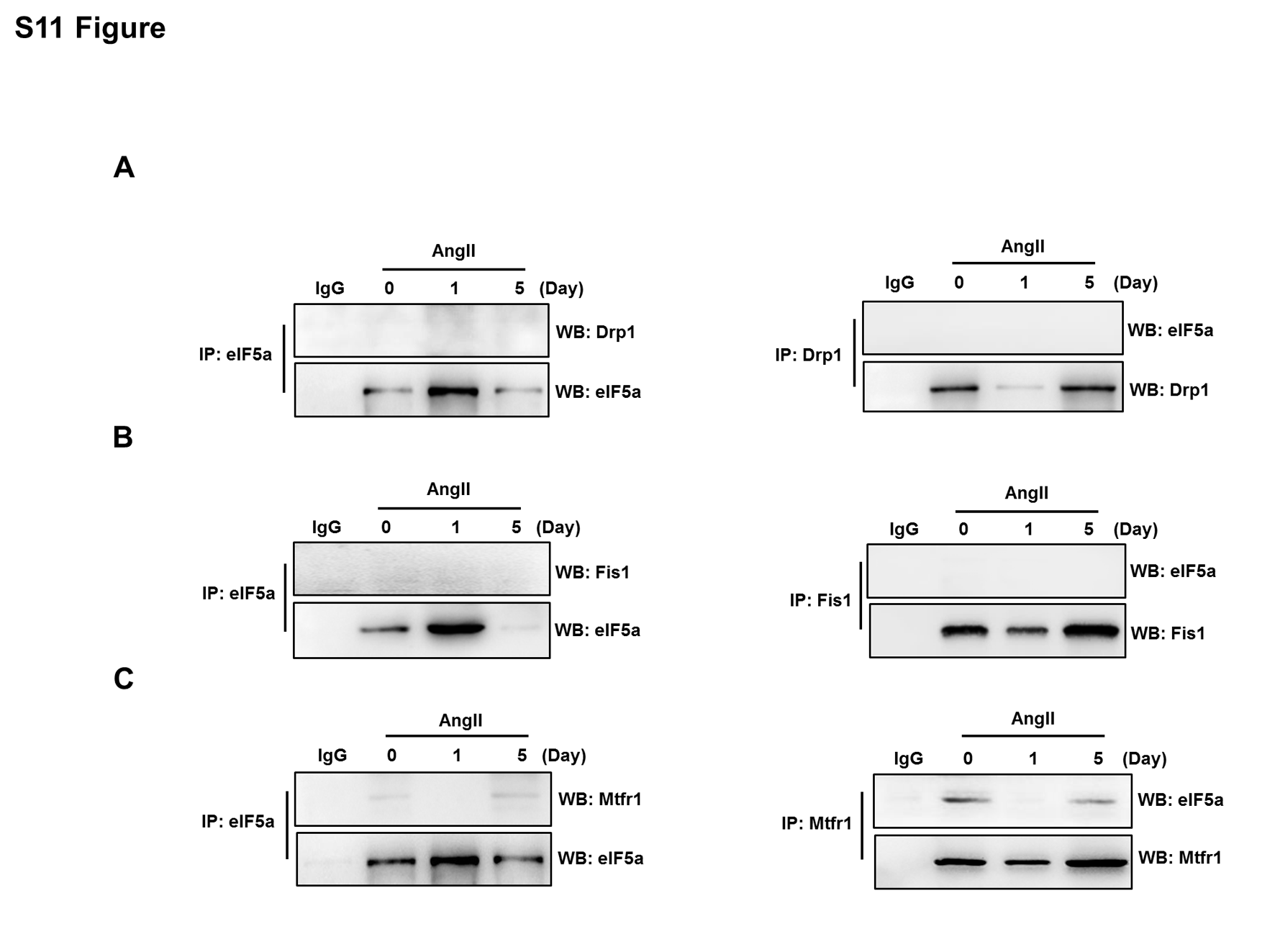

Supplement: S11 Fig — (A-C) Reciprocal co-immunoprecipitation assay for interaction between eIF5a and Drp1 (A), Fis1 (B), or Mtfr1 (C) in VSMCs treated with Ang II (100 nmol/L) for the indicated times. IgG was used as a negative control. For raw immunoblots, please see S1 Blots. Ang II, angiotensin II; Drp1, dynamin-related protein 1; eIF5a, eukaryotic translation initiation factor 5a; Fis1, fission mitochondrial 1; IgG, immunoglobulin G; Mtfr1, mitochondrial fission regulator 1; VSMC, vascular smooth muscle cell. (TIF) [file pbio.3000808.s011.tif]

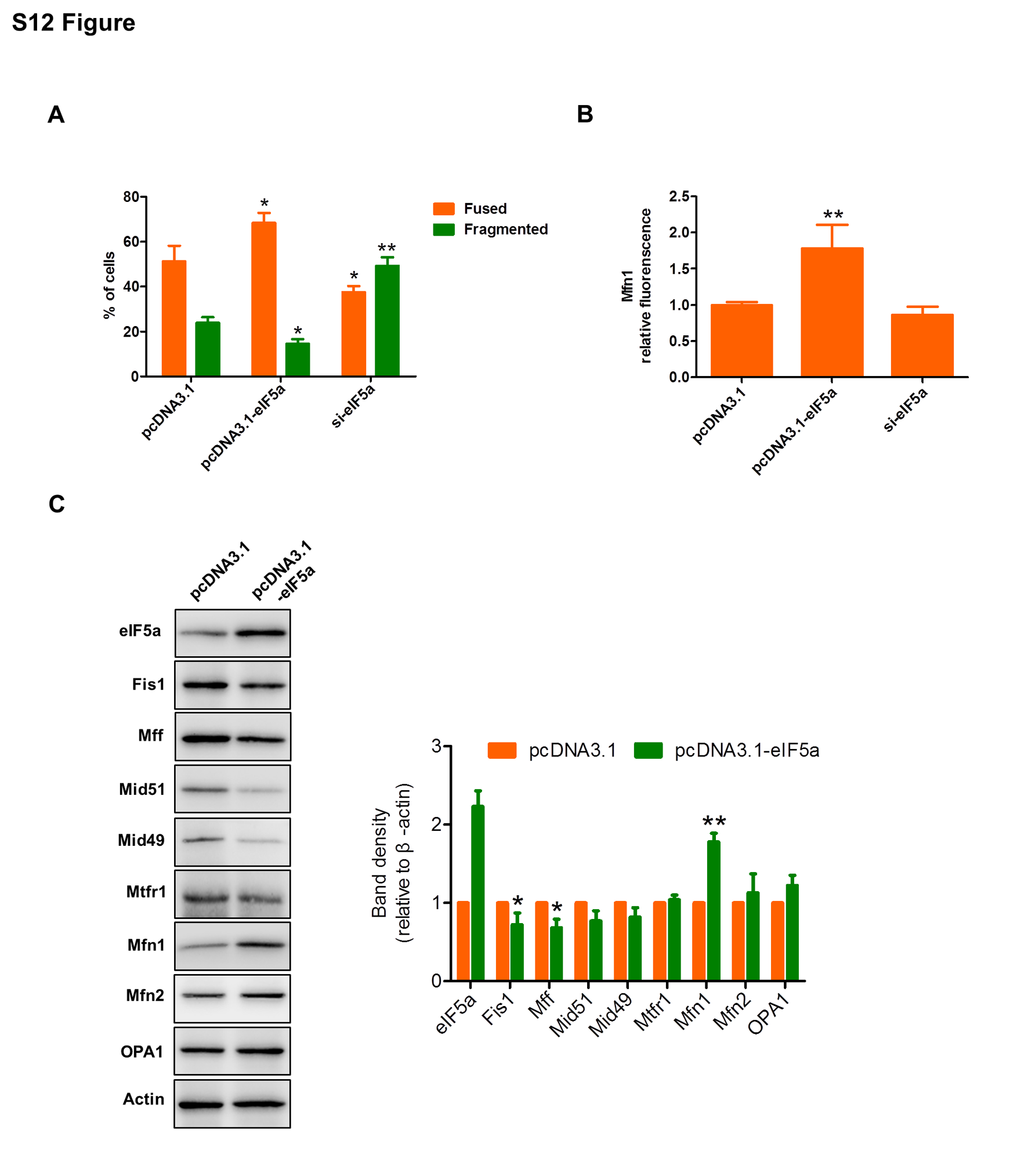

Supplement: S12 Fig — (A) The percentage of cells containing fused and fragmented mitochondria in Fig 6C. *P < 0.05 versus pcDNA3.1. (B) Relative fluorescence intensity of Mfn1 in Fig 6C. Data represent the mean ± SD. **P < 0.01 versus pcDNA3.1. (C) Representative western blot image of eIF5a, Fis1, Mff, Mid51, Mid49, Mtfr1, Mfn1, Mfn2, and OPA1 in mouse VSMCs transfected with pcDNA3.1 or pcDNA3.1-eIF5a. Right: Band intensities that were measured and normalized to β-actin. Data represent the mean ± SD. *P < 0.05 and **P < 0.01 versus pcDNA3.1. For numerical raw data, please see S1 Data. For raw immunoblots, please see S1 Blots. eIF5a, eukaryotic translation initiation factor 5a; Fis1, fission mitochondrial 1; Mff, mitochondrial fission factor; Mfn1, mitofusin 1; Mfn2, mitofusin 2; Mid49, mitochondrial dynamics protein 49; Mid51, mitochondrial dynamics protein 51; Mtfr1, mitochondrial fission regulator 1; OPA1, Optic atrophy type 1; VSMC, vascular smooth muscle cell. (TIF) [file pbio.3000808.s012.tif]

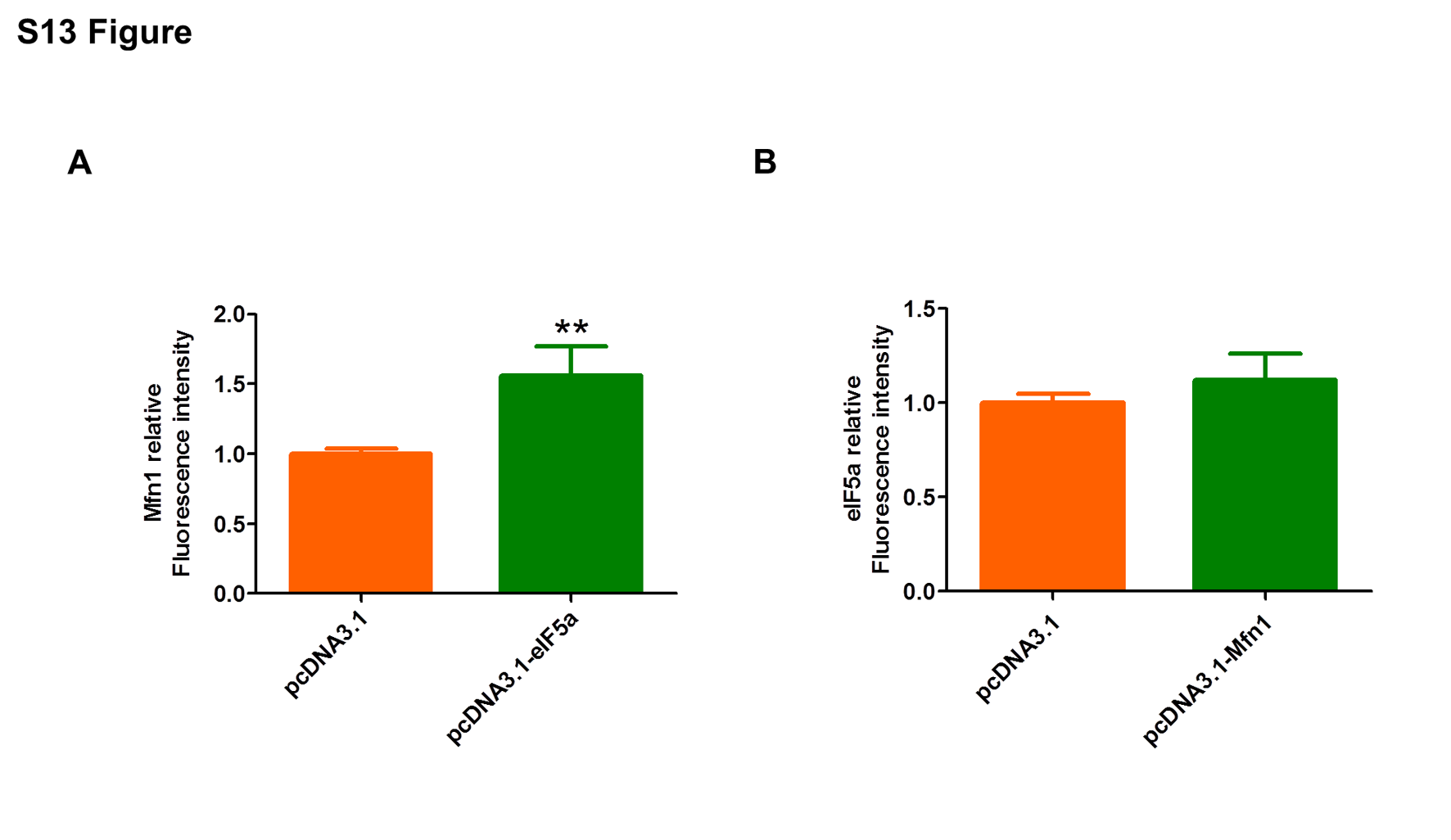

Supplement: S13 Fig — (A, B) Statistic analysis of relative fluorescence intensity of Mfn1 and eIF5a in Fig 6F (A) and Fig 6G (B), respectively. Data represent the mean ± SD. **P < 0.01 versus pcDNA3.1. For numerical raw data, please see S1 Data. eIF5a, eukaryotic translation initiation factor 5a; Mfn1, mitofusin 1. (TIF) [file pbio.3000808.s013.tif]

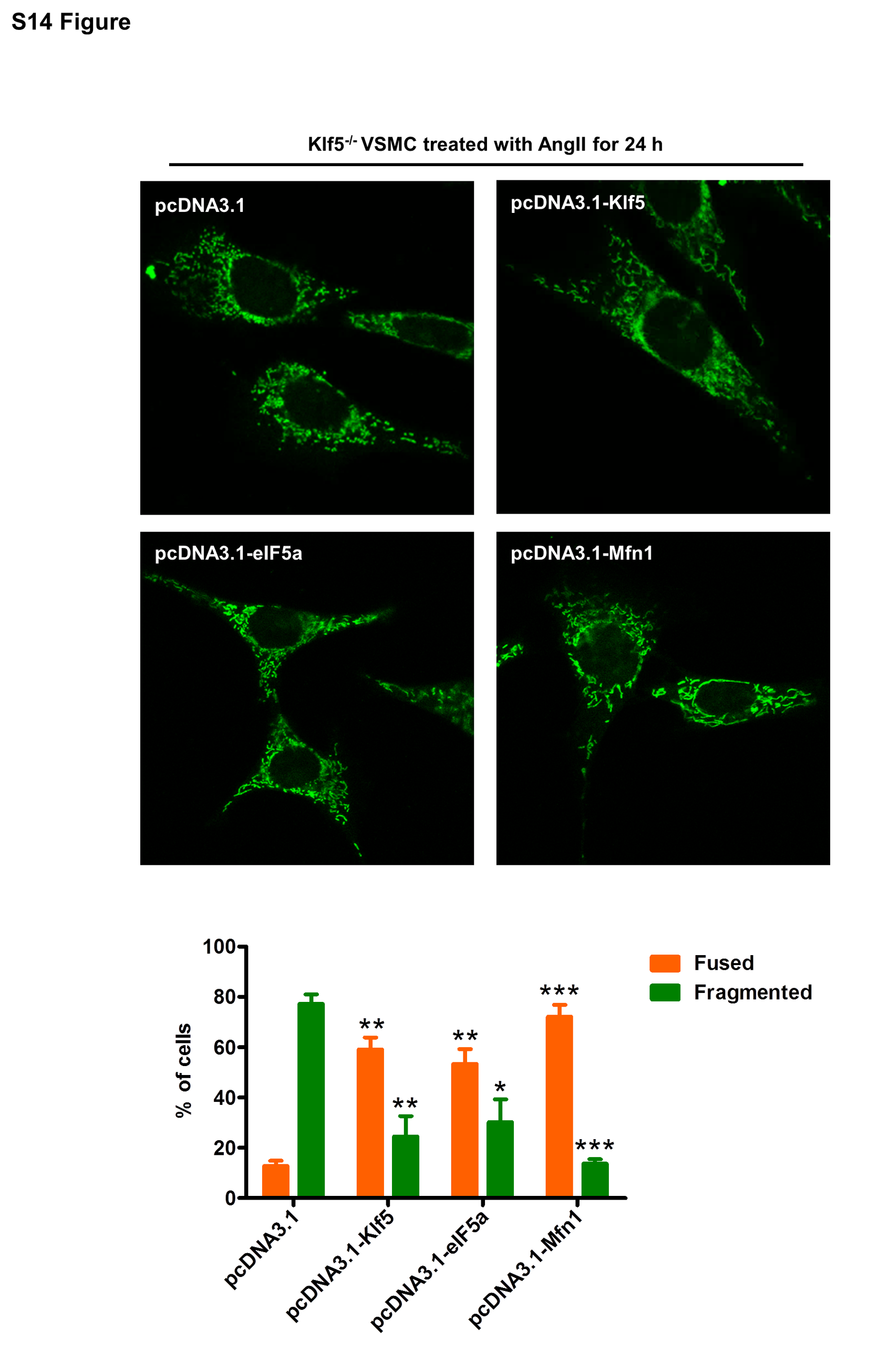

Supplement: S14 Fig — MitoTracker green staining detects the mitochondrial morphology in mouse VSMCs transfected with expression plasmids encoding Klf5, elF5a, or Mfn1. Down: The percentage of cells containing fused and fragmented mitochondria was quantified from more than 100 cells. Scale bars = 10 μm. Data represent mean ± SEM, *P < 0.05, **P < 0.01, and ***P < 0.001 versus pcDNA3.1. For numerical raw data, please see S1 Data. elF5a, eukaryotic translation initiation factor; Klf5, Krüppel-like factor 5; Mfn1, mitofusin 1; VSMC, vascular smooth muscle cell. (TIF) [file pbio.3000808.s014.tif]

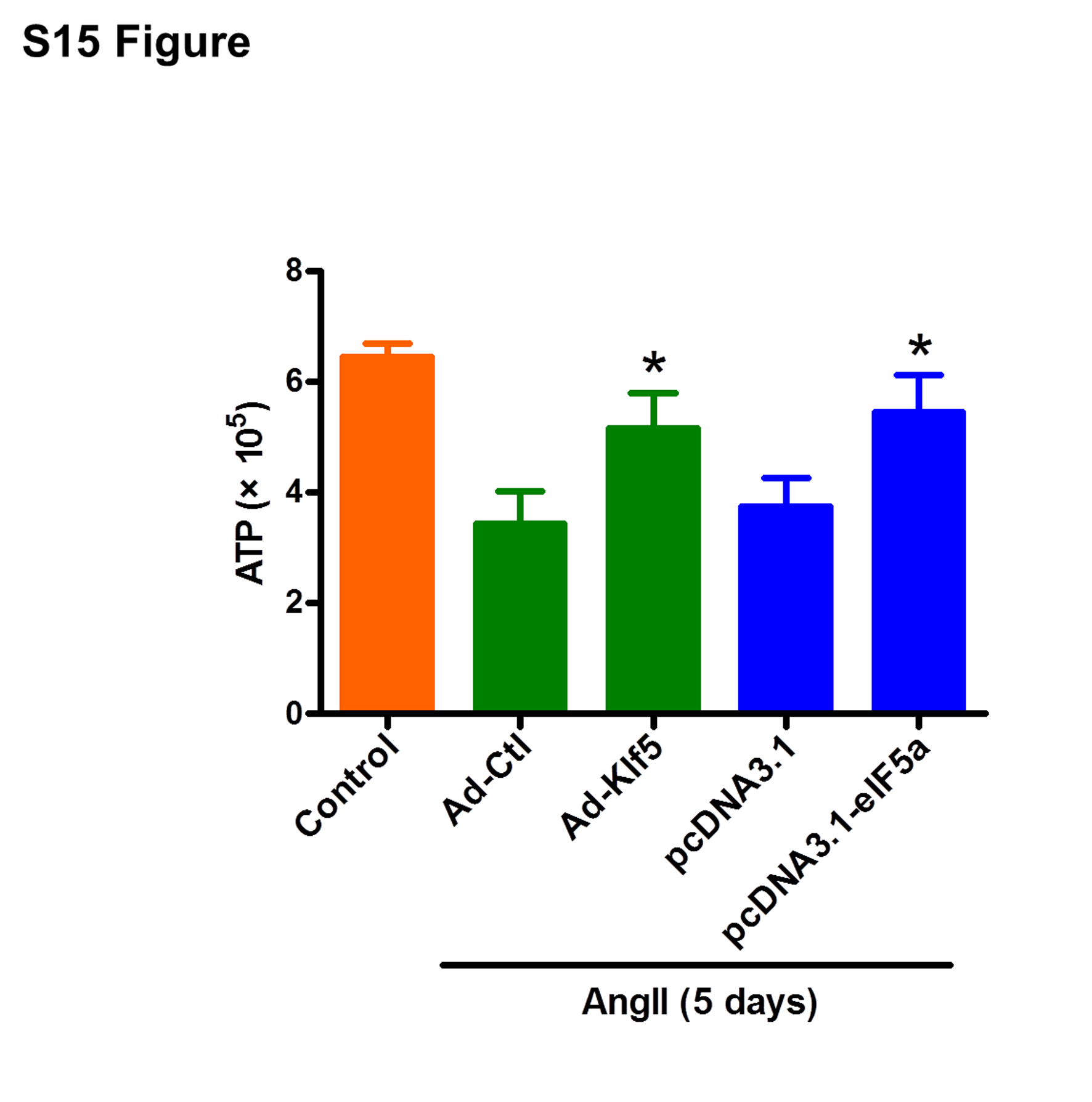

Supplement: S15 Fig — ATP content in mouse VSMCs infected or transfected with Ad-Klf5 or pcDNA3.1-eIF5a and then treated with Ang II for 5 days. Mouse VSMCs were infected or transfected with Ad-Klf5 or pcDNA3.1-eIF5a and then treated with Ang II for 5 days. ATP levels were determined using the Adenosine 5′-triphosphate (ATP) Bioluminescent Assay Kit (Sigma) following the manufacturer’s instructions. Data represent mean ± SEM, *P < 0.05 versus Ad-Ctl or pcDNA3.1. For numerical raw data, please see S1 Data. Ad-Ctl, adenoviruses encoding control; Ad-Klf5, adenoviruses encoding Klf5; Ang II, angiotensin II; eIF5a, eukaryotic translation initiation factor 5a; VSMC, vascular smooth muscle cell. (TIF) [file pbio.3000808.s015.tif]

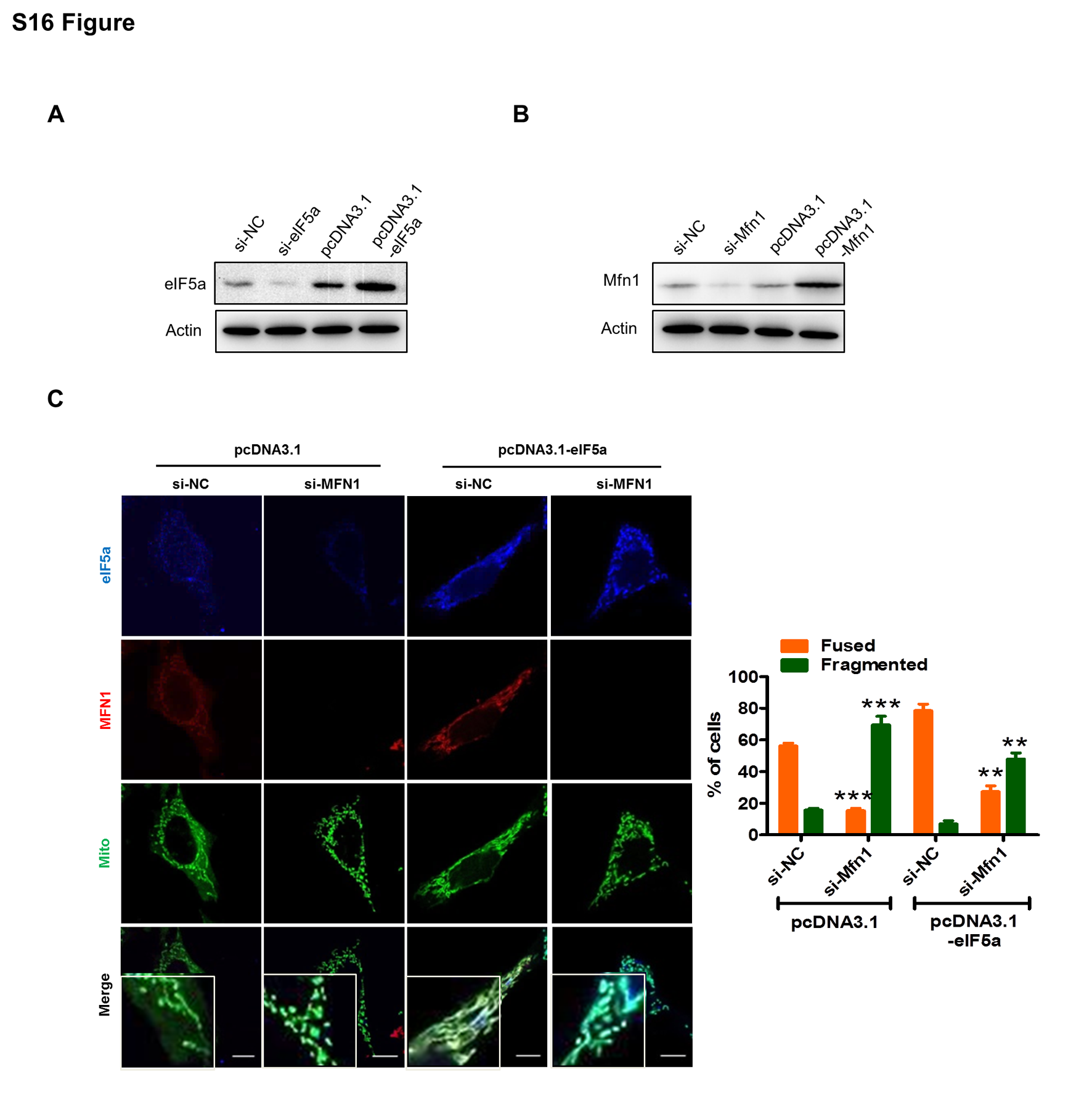

Supplement: S16 Fig — (A,B) Mouse VSMCs were transfected with si-NC, si-eIF5a, pcDNA3.1, or pcDNA3.1-eIF5a (A), as well as with si-NC, si-Mfn1, pcDNA3.1, or pcDNA3.1-Mfn1 (B) for 36 hours. Crude proteins were extracted and then subjected to western blotting with anti-eIF5a or anti-Mfn1 antibodies. β-actin was used as a loading control. (C) Mouse VSMCs were co-transfected with pcDNA3.1 or pcDNA3.1-eIF5a and si-NC or si-Mfn1 for 36 hours. Co-localization of eIF5a with Mfn1 and Mitotracker was detected by confocal microscopy. Scale bars = 10 μm. The percentage of cells containing fragmented and fused mitochondria was quantified from more than 300 cells. Data are expressed as mean ± SEM. **P < 0.01 or ***P < 0.001 versus si-NC. For numerical raw data, please see S1 Data. For raw immunoblots, please see S1 Blots. eIF5a, eukaryotic translation initiation factor 5a; Mfn1, mitofusin 1; si-eIF5a, short interfering RNA targeting eIF5a; si-Mfn1, short interfering RNA targeting Mfn1; si-NC, short interfering RNA negative control; VSMC, vascular smooth muscle cell. (TIF) [file pbio.3000808.s016.tif]

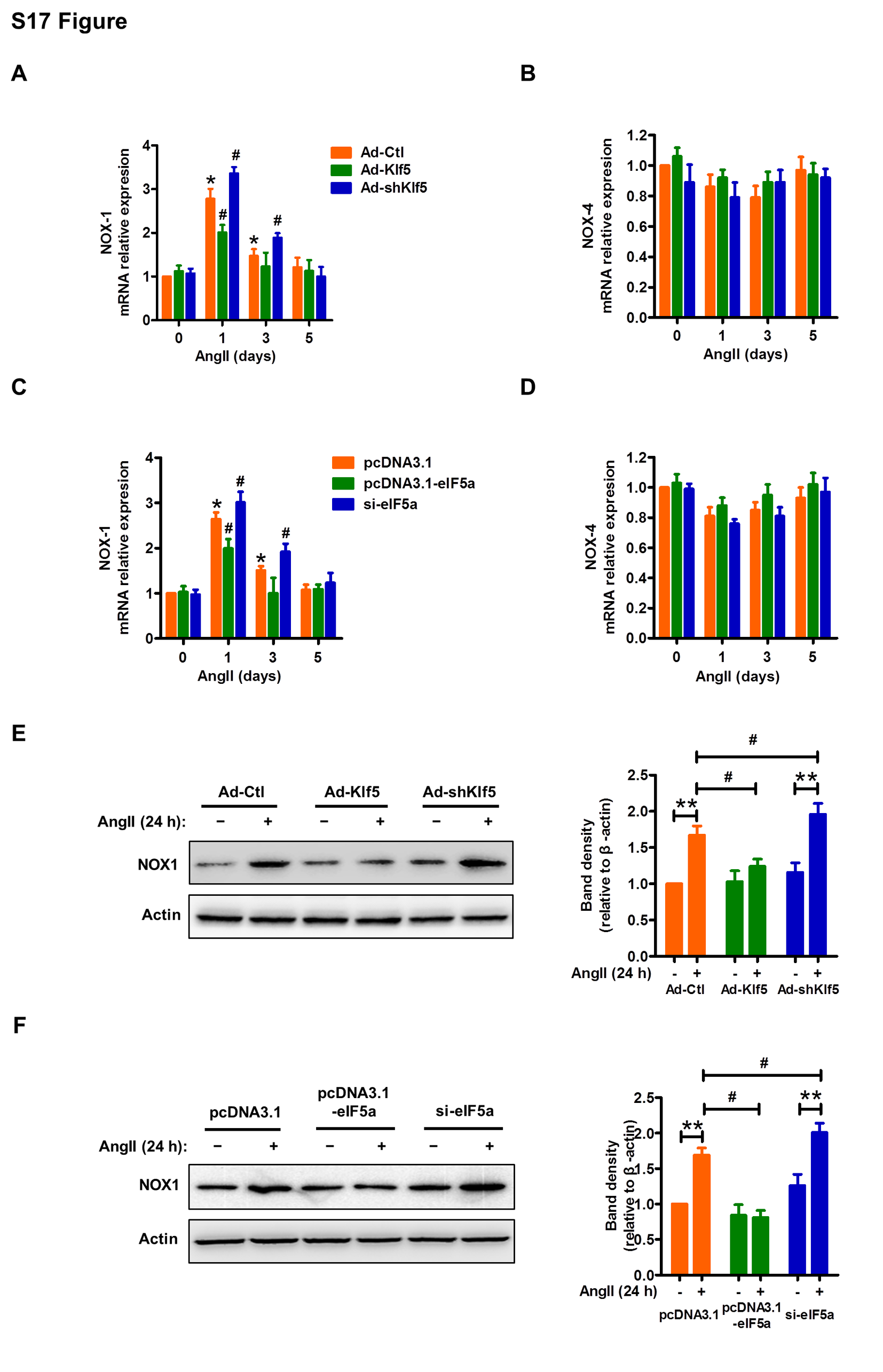

Supplement: S17 Fig — (A-D) Mouse VSMCs were infected or transfected with Ad-Klf5 and Ad-shKlf5 (A,B) or pcDNA3.1-eIF5a and si-eIF5a (C,D) and then treated with Ang II for the indicated times. NOX1 and NOX4 mRNA expression was quantified using qRT-PCR, and normalized to GAPDH and expressed as fold increase over day 0. (E,F) Klf5-overexpressing or knocking down (E) and eIF5a-overexpressing or knocking down (F) mouse VSMCs were treated with Ang II for 24 hours. Crude proteins were extracted and then subjected to western blotting with anti-NOX1 antibody. β-actin was used as a loading control. Right: Band intensities that were measured and normalized to β-actin. Data represent the mean ± SD. *P < 0.05 and **P < 0.01 versus 0 day, #P < 0.05 versus Ad-Ctl and pcDNA3.1 at the same day or at the Ang II treatment. For numerical raw data, please see S1 Data. For raw immunoblots, please see S1 Blots. Ad-Ctl, adenoviruses encoding control; Ad-Klf5, adenoviruses encoding Klf5; Ad-shKlf5, adenoviruses encoding small hairpin Klf5; Ang II, angiotensin II; eIF5a, eukaryotic translation initiation factor 5a; GAPDH, glyceraldehyde-3-phosphate dehydrogenase; Klf5, Krüppel-like factor 5; NOX1, NAPDH oxidase 1; qRT-PCR, quantitative real-time PCR; si-eIF5a, short interfering RNA targeting eIF5a; VSMC, vascular smooth muscle cell. (TIF) [file pbio.3000808.s017.tif]

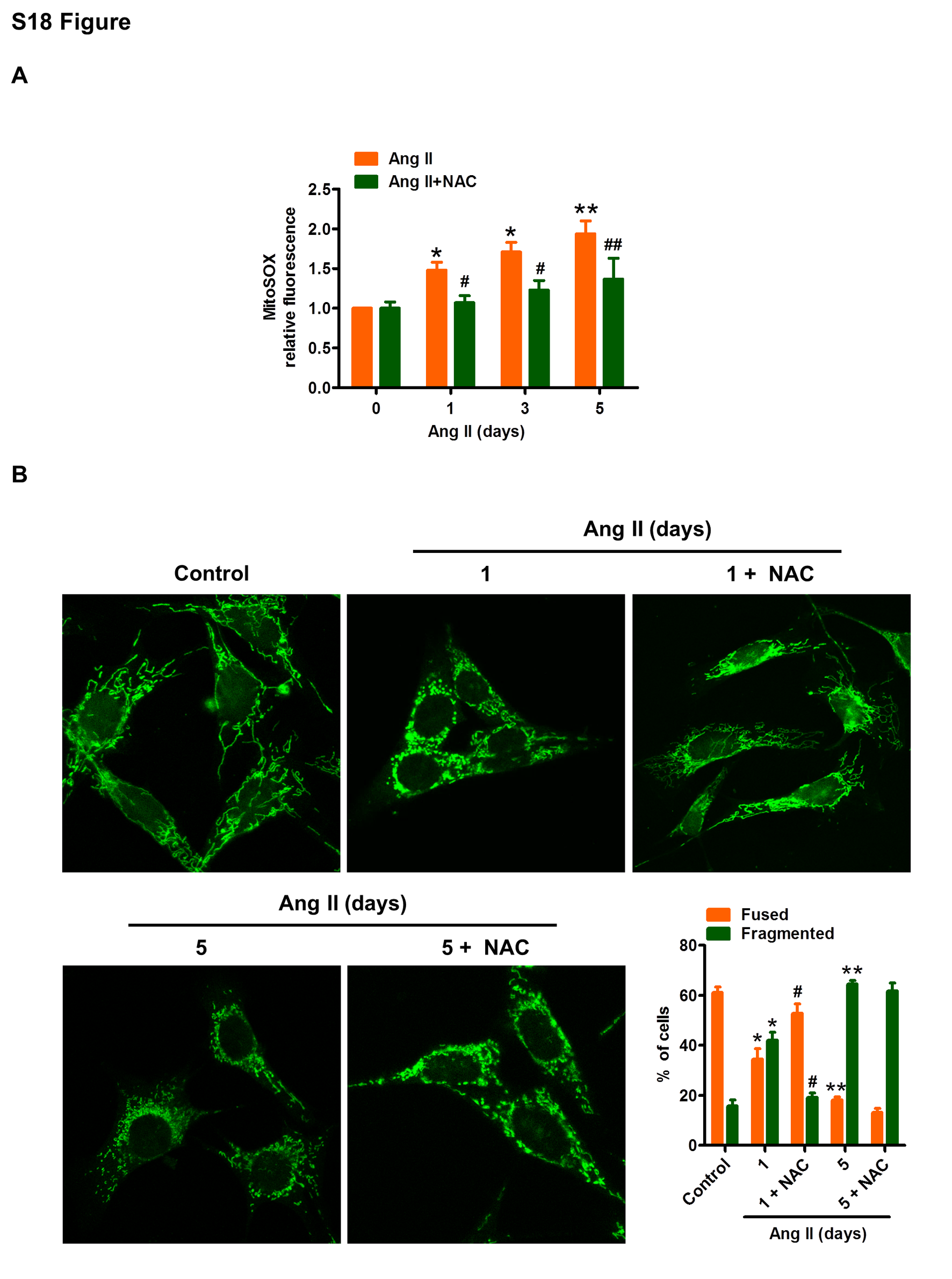

Supplement: S18 Fig — (A) Mouse VSMCs pretreated with NAC (500 μM) were treated or not with Ang II for the indicated times. Quantification of MitoSox fluorescence of VSMCs by FACS analysis using Mitosox Green dye. (B) NAC-pretreated VSMCs were stimulated with Ang II for 1 or 5 days, and then subjected to MitoTracker Green staining to visualize mitochondrial morphology. Right: The percentage of cells containing fragmented and fused mitochondria was quantified from more than 100 cells. All data represent the mean ± SEM. *P < 0.05 and **P < 0.01 versus 0 day, #P < 0.05 and ##P < 0.01 versus untreated with NAC at the same day. For numerical raw data, please see S1 Data. Ang II, angiotensin II; FACS, flow analysis of cytosorting; NAC, N-Acetyl-L-cysteine; VSMC, vascular smooth muscle cell. (TIF) [file pbio.3000808.s018.tif]

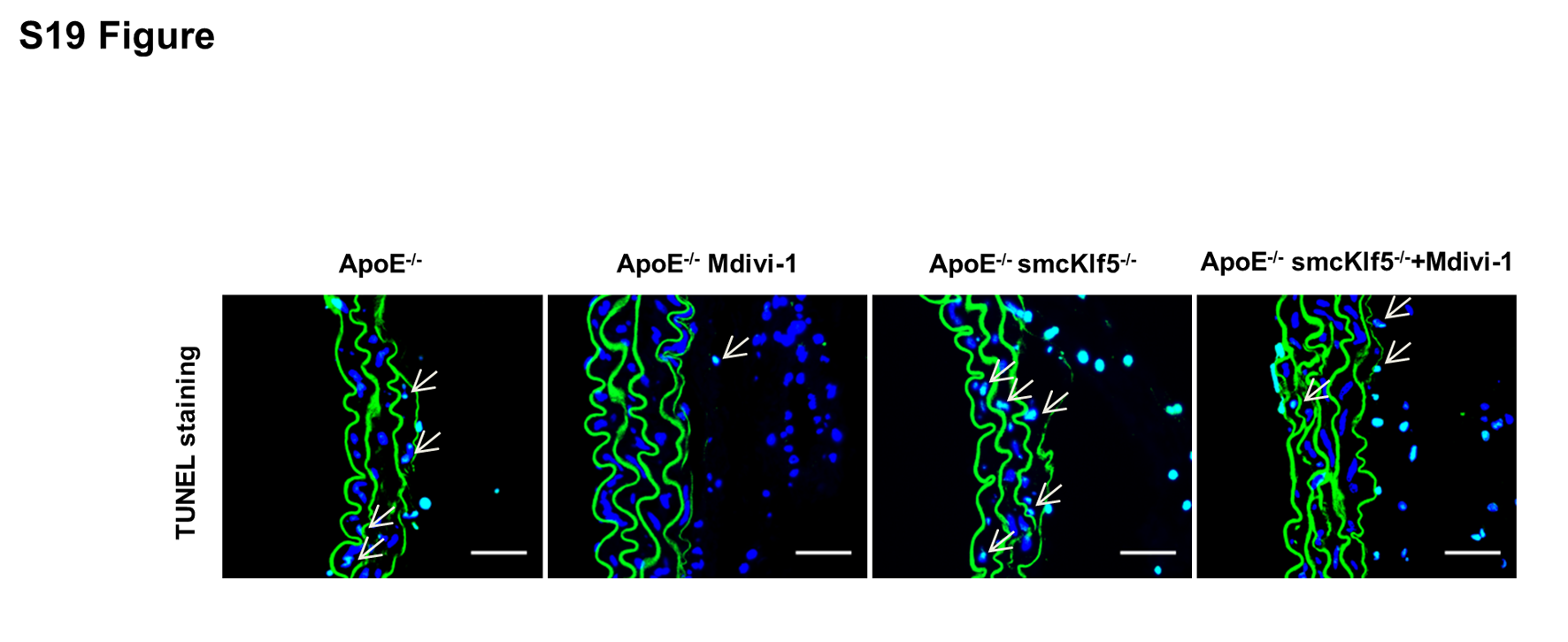

Supplement: S19 Fig — Mdivi-1 (1.2 mg/kg) was administrated to mice intraperitoneally 15 minutes before the onset of Ang II perfusion and then injected once a week for 4 weeks. Representative TUNEL-stained (green) and DAPI-stained (blue) slices show cell apoptosis in the abdominal aortas of ApoE−/− and ApoE−/− smcKlf5−/− mice. Scale bars = 50 μm. Ang II, angiotensin II; Mdivi-1, mitochondrial division inhibitor 1. (TIF) [file pbio.3000808.s019.tif]
