# Supplementary material for: Klf5 down-regulation induces vascular senescence through eIF5a depletion and mitochondrial fission
Source: PLoS Biol. 2020 Aug 20;18(8):e3000808. doi: 10.1371/journal.pbio.3000808 (PMC7462304; doi:10.1371/journal.pbio.3000808)

Full blot images-Figure 1A

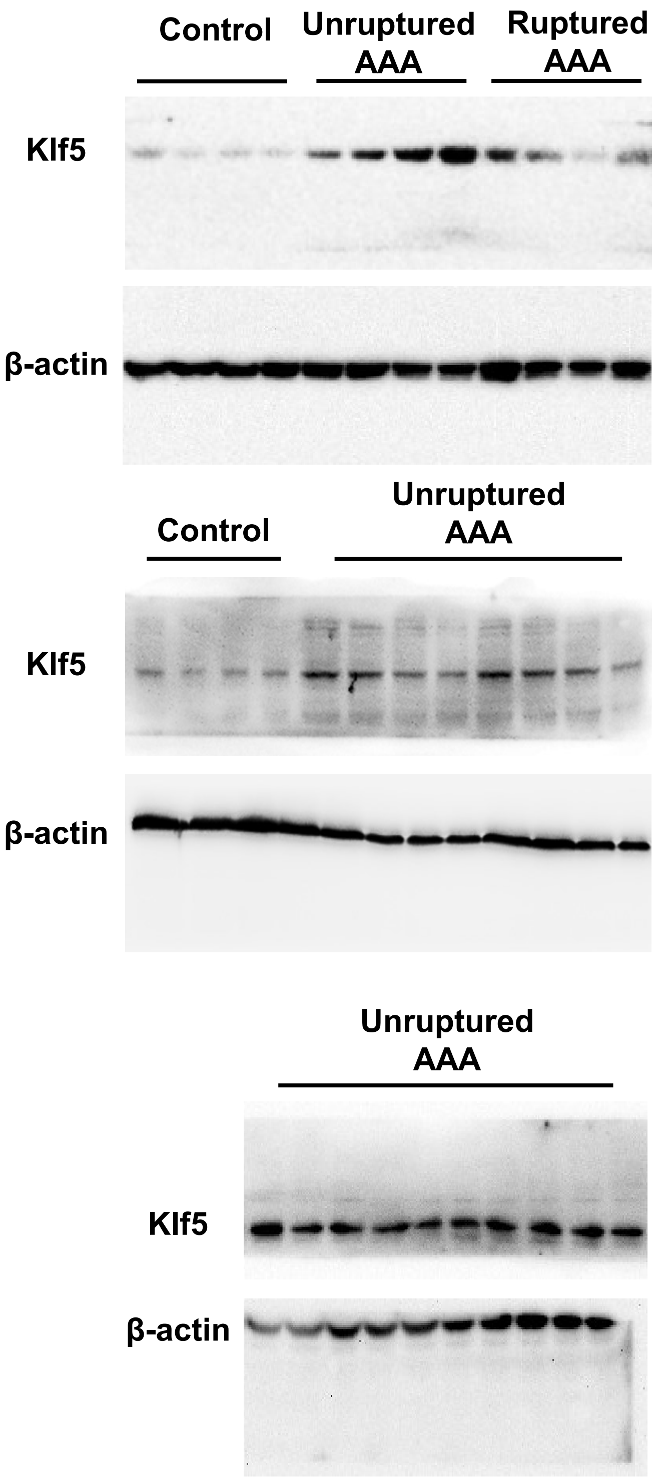

Full blot images-Figure 1H

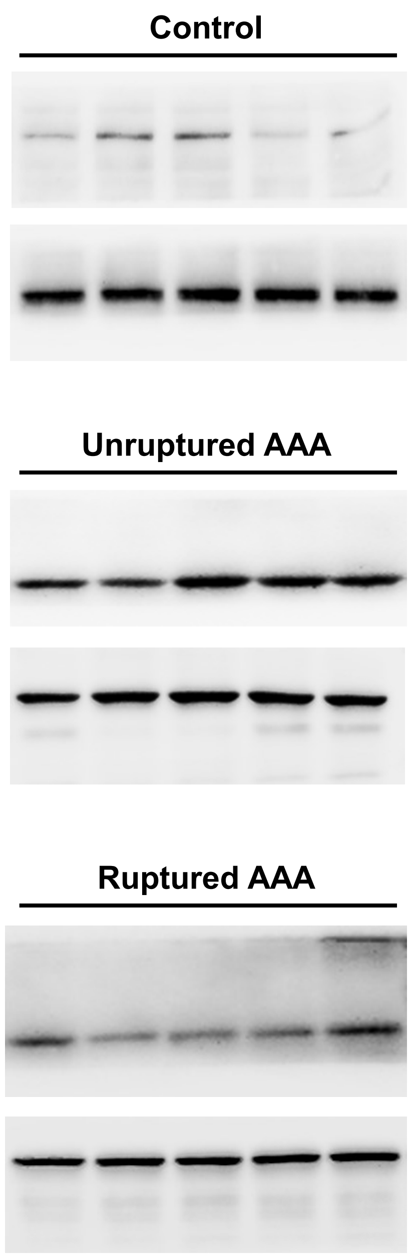

Full blot images-Figure 2K

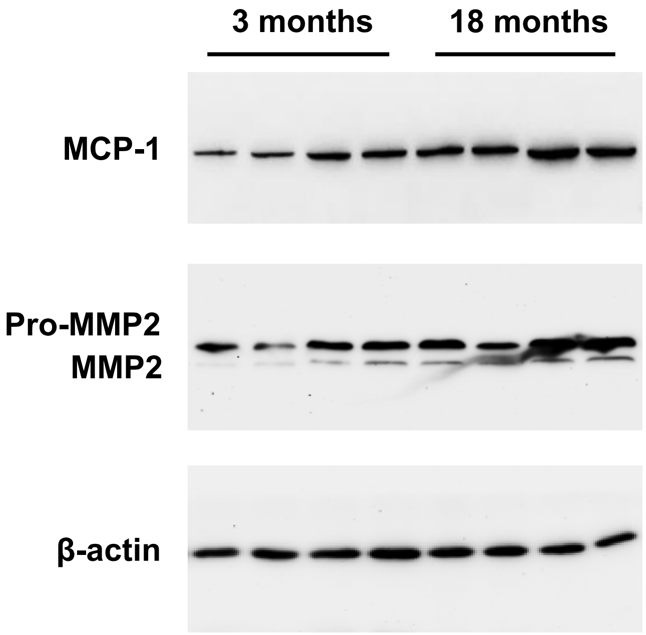

Full blot images-Figure 3C

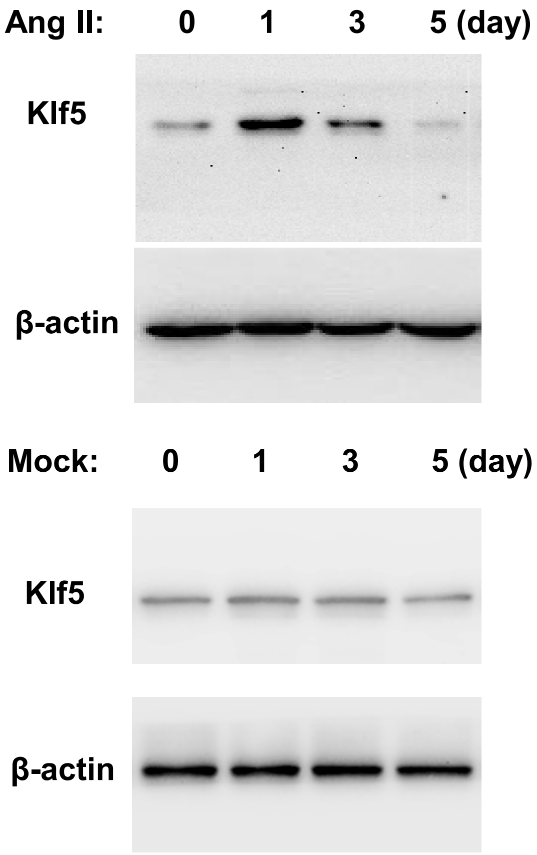

Full blot images-Figure 4E

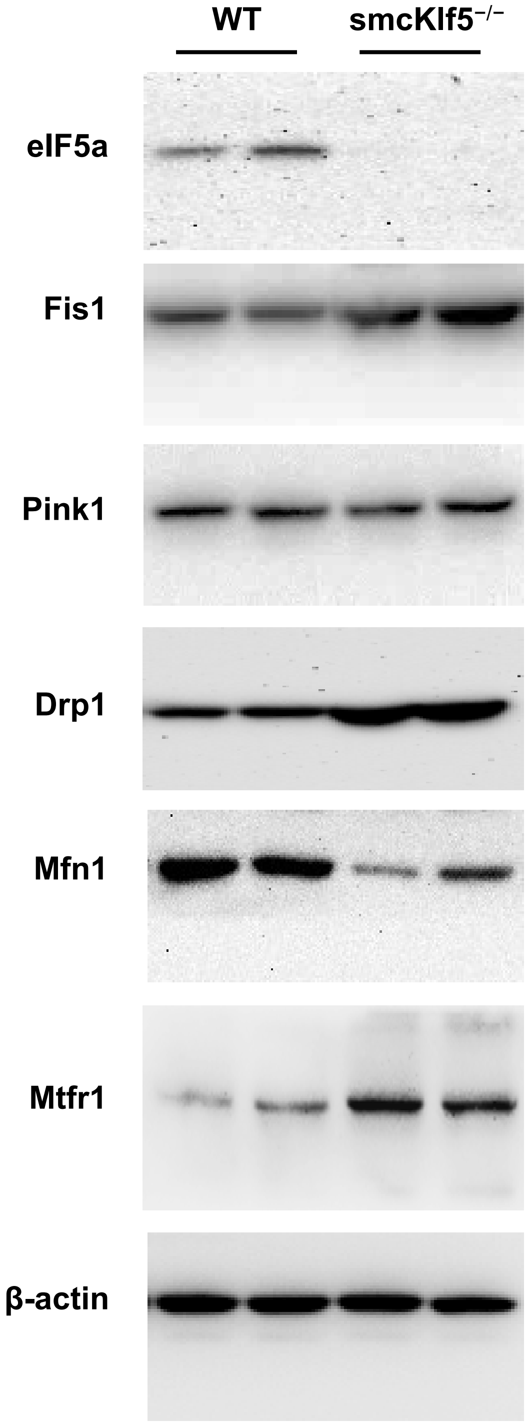

Full blot images-Figure 4F

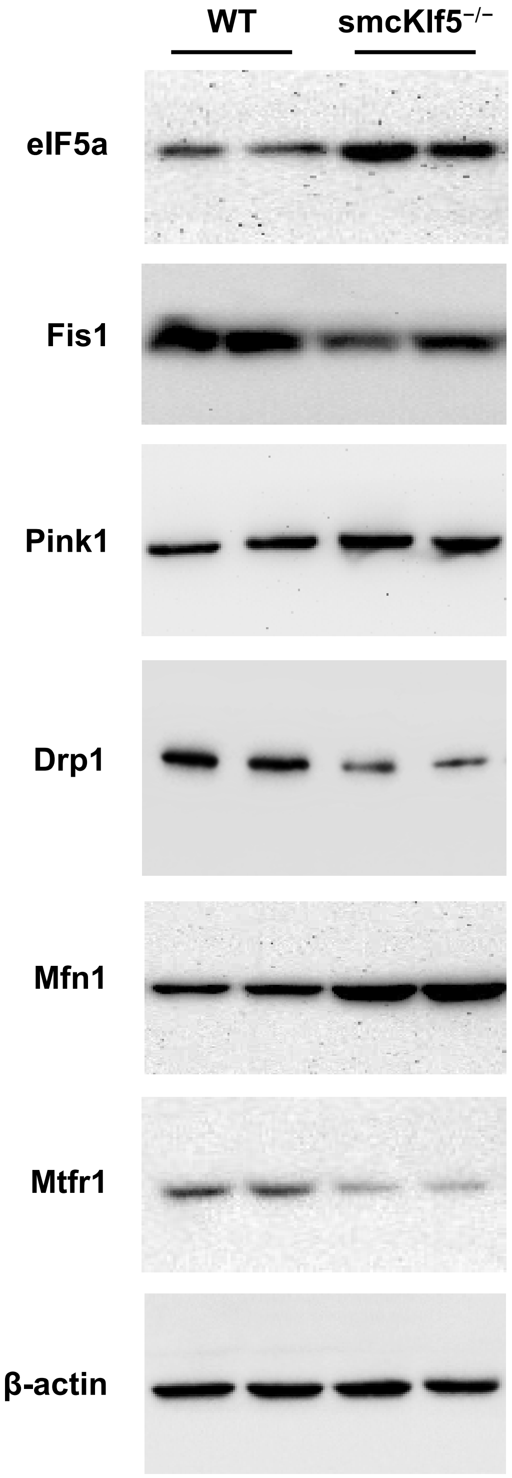

Full blot images-Figure 5A

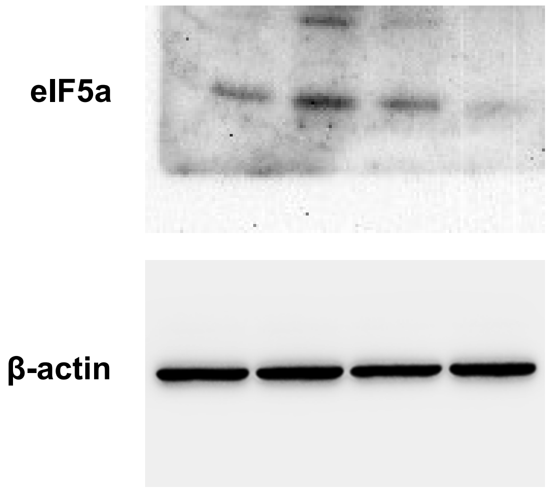

Full blot images-Figure 5D

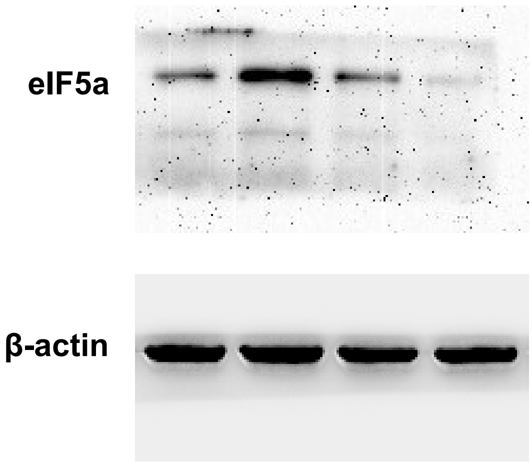

Full blot images-Figure 5H

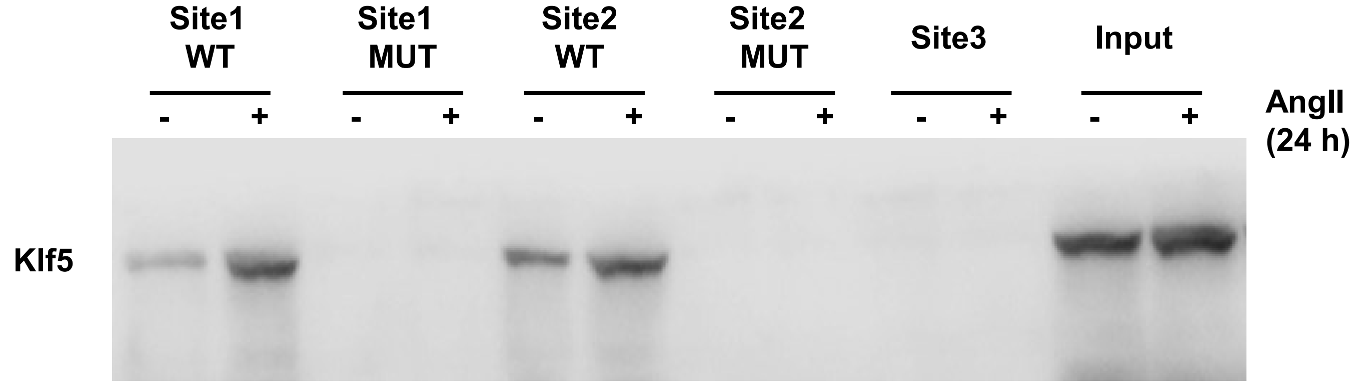

Full blot images-Figure 6A

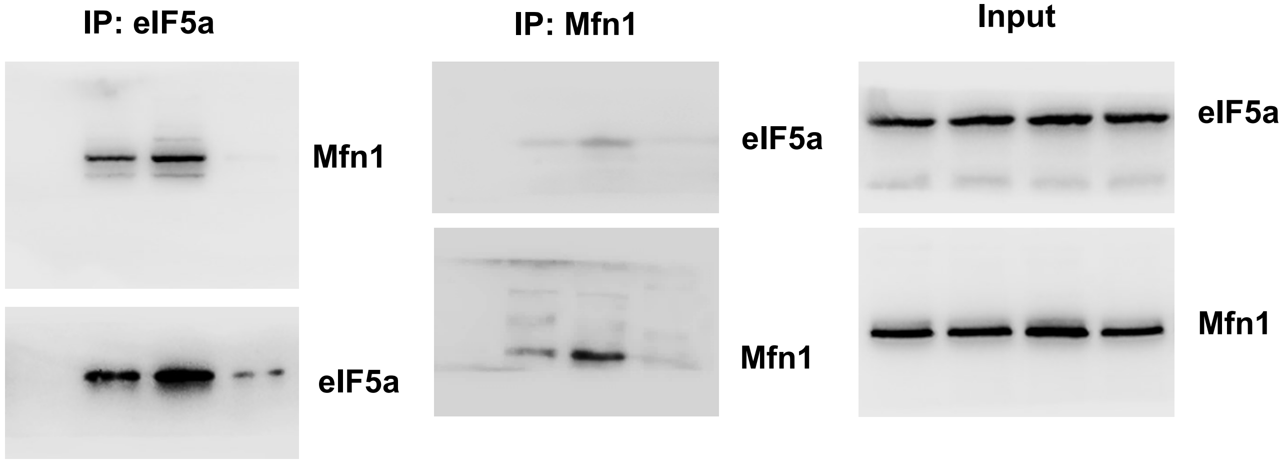

Full blot images-Figure S3B

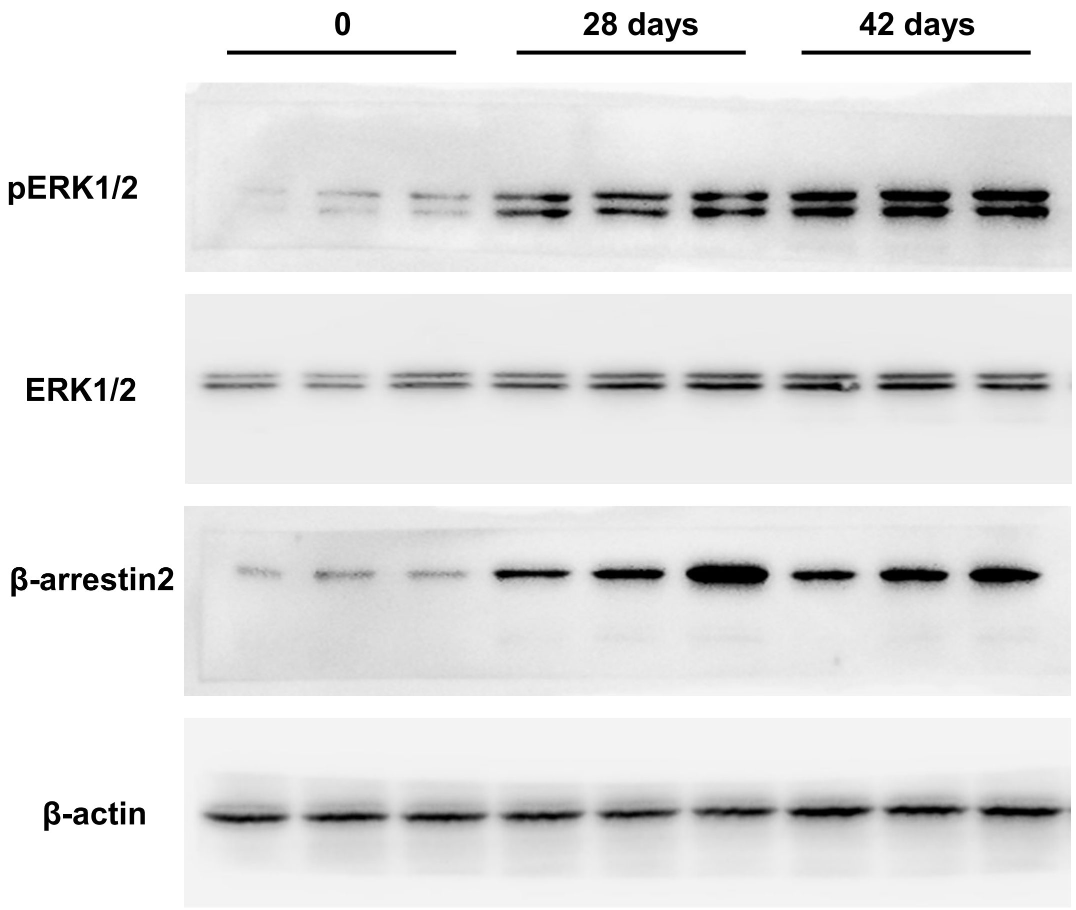

Full blot images-Figure S3C

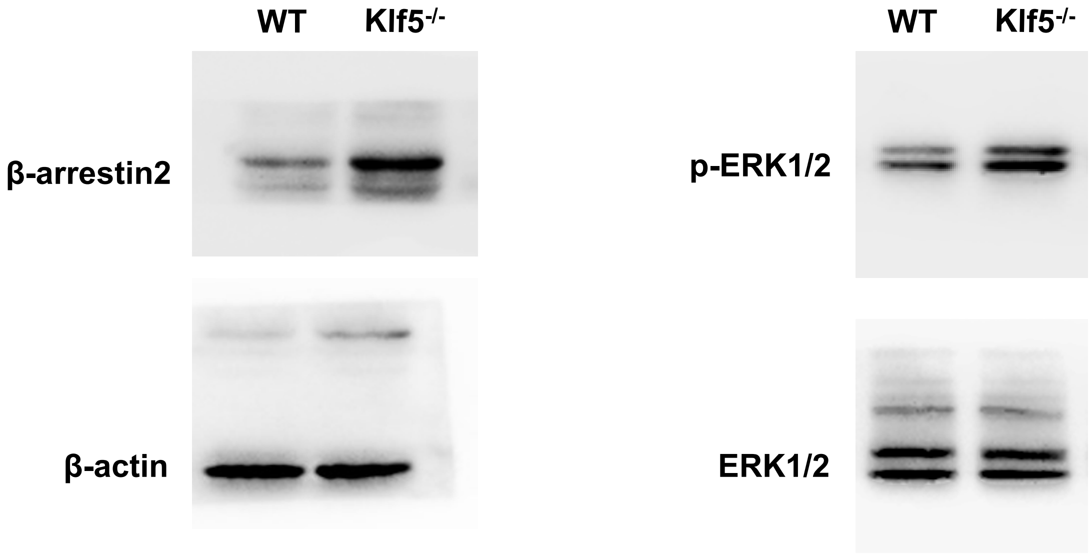

Full blot images-Figure S8A

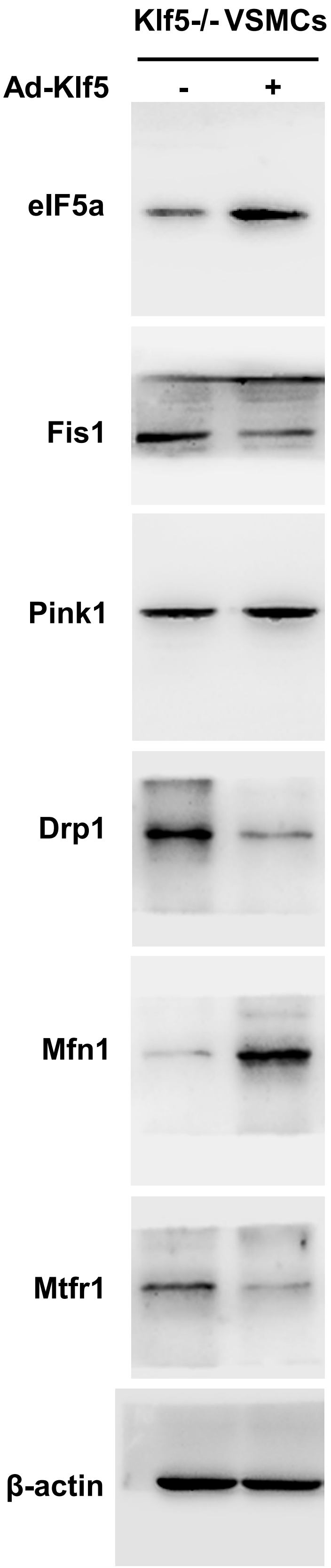

Full blot images-Figure S8B

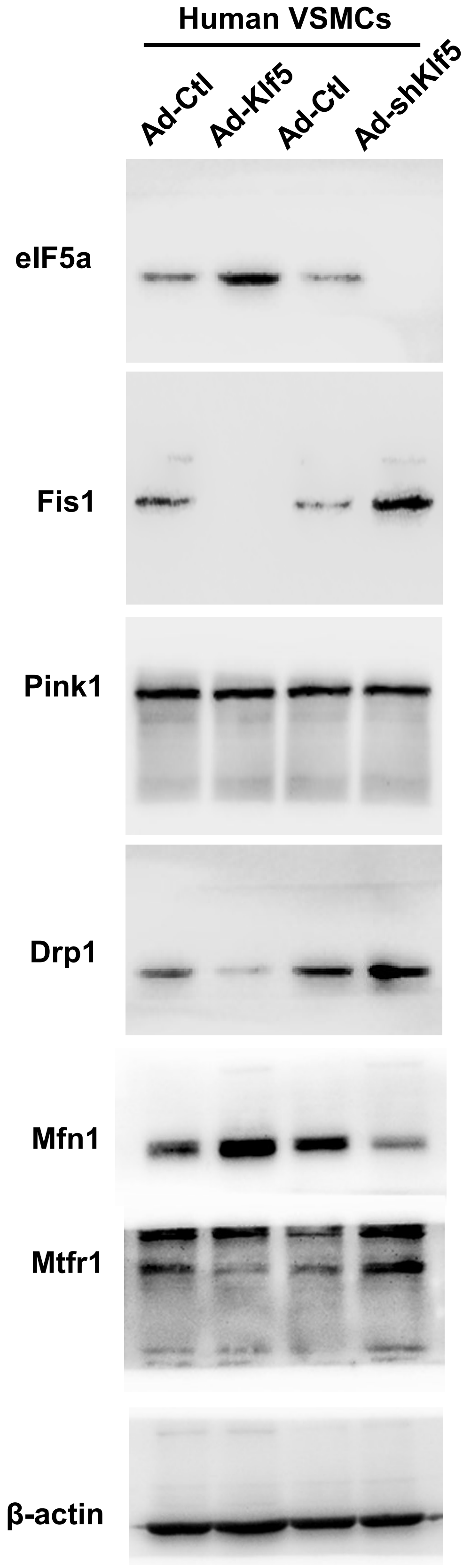

Full blot images-Figure S9A

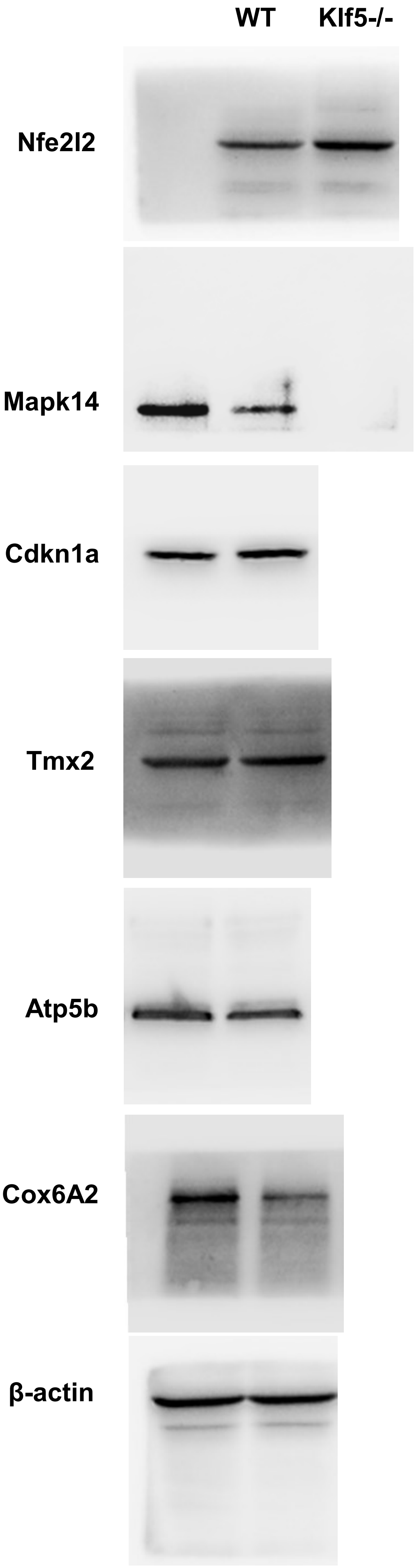

Full blot images-Figure S9B

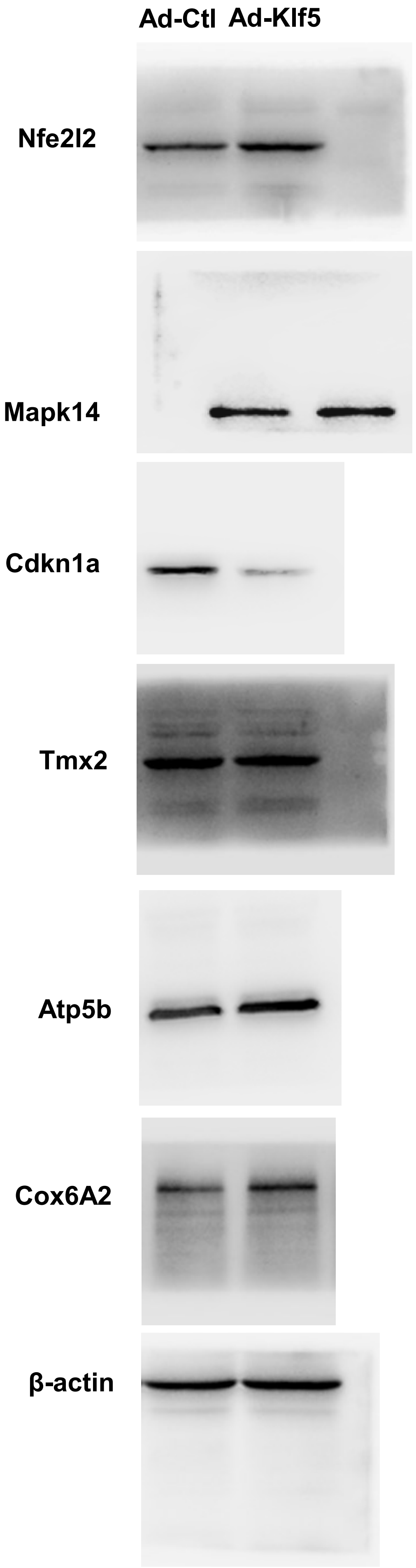

Full blot images-Figure S10H

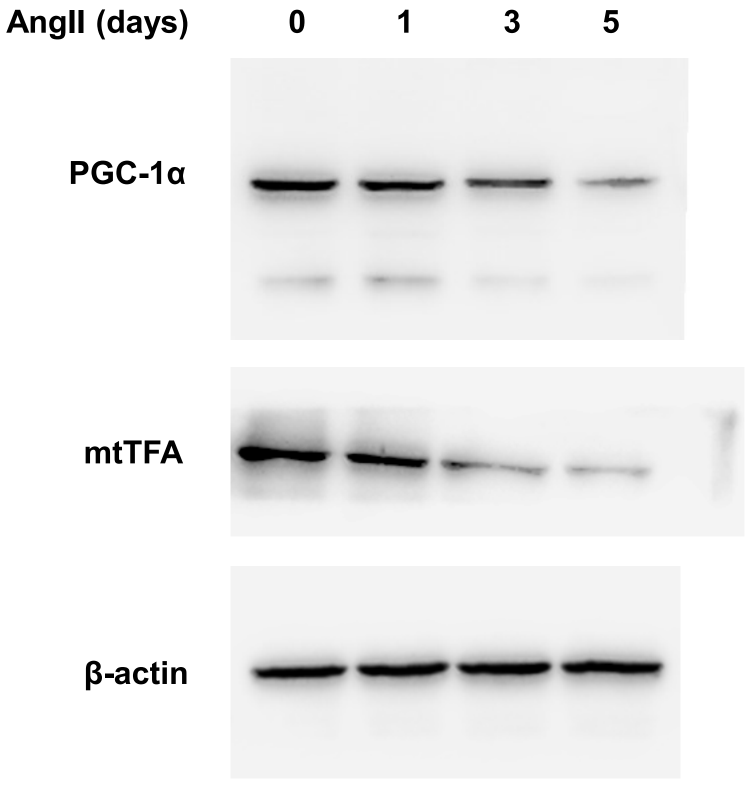

Full blot images-Figure S11A

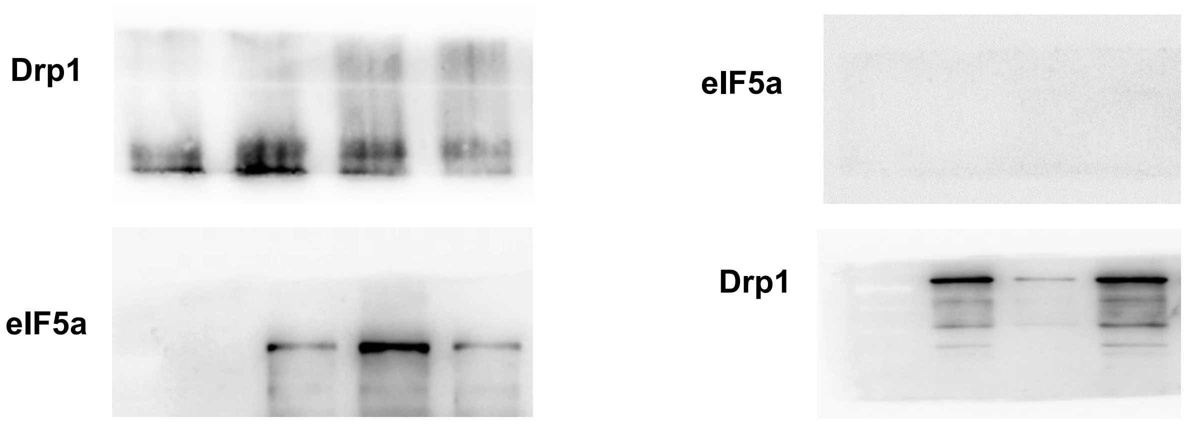

Full blot images-Figure S11B

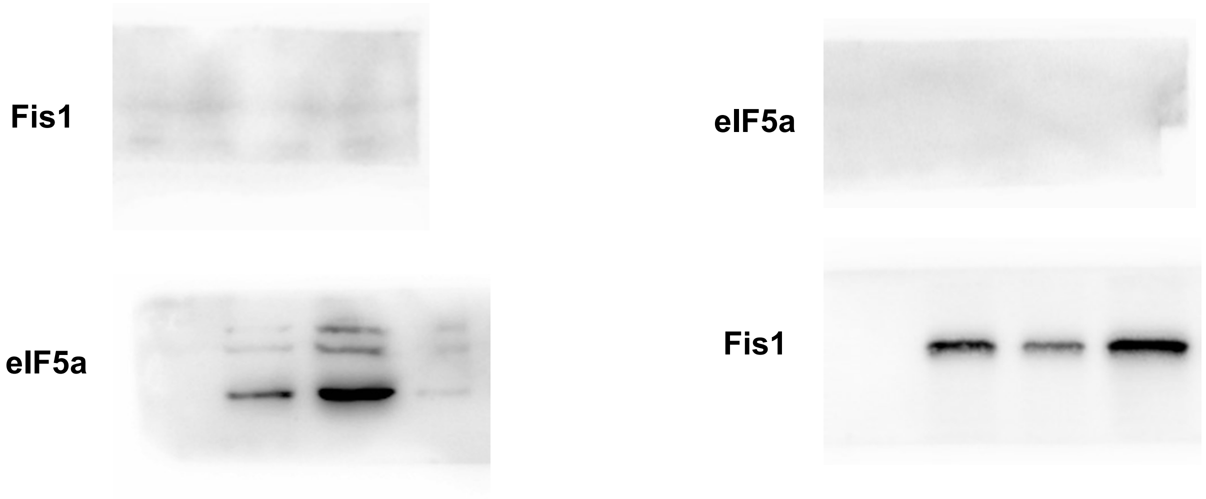

Full blot images-Figure S11C

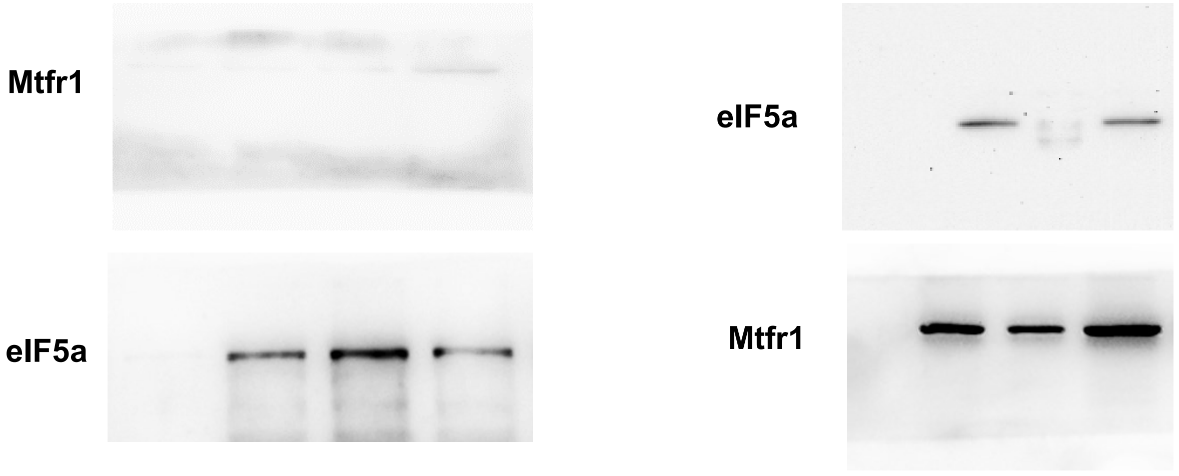

Full blot images-Figure S12C

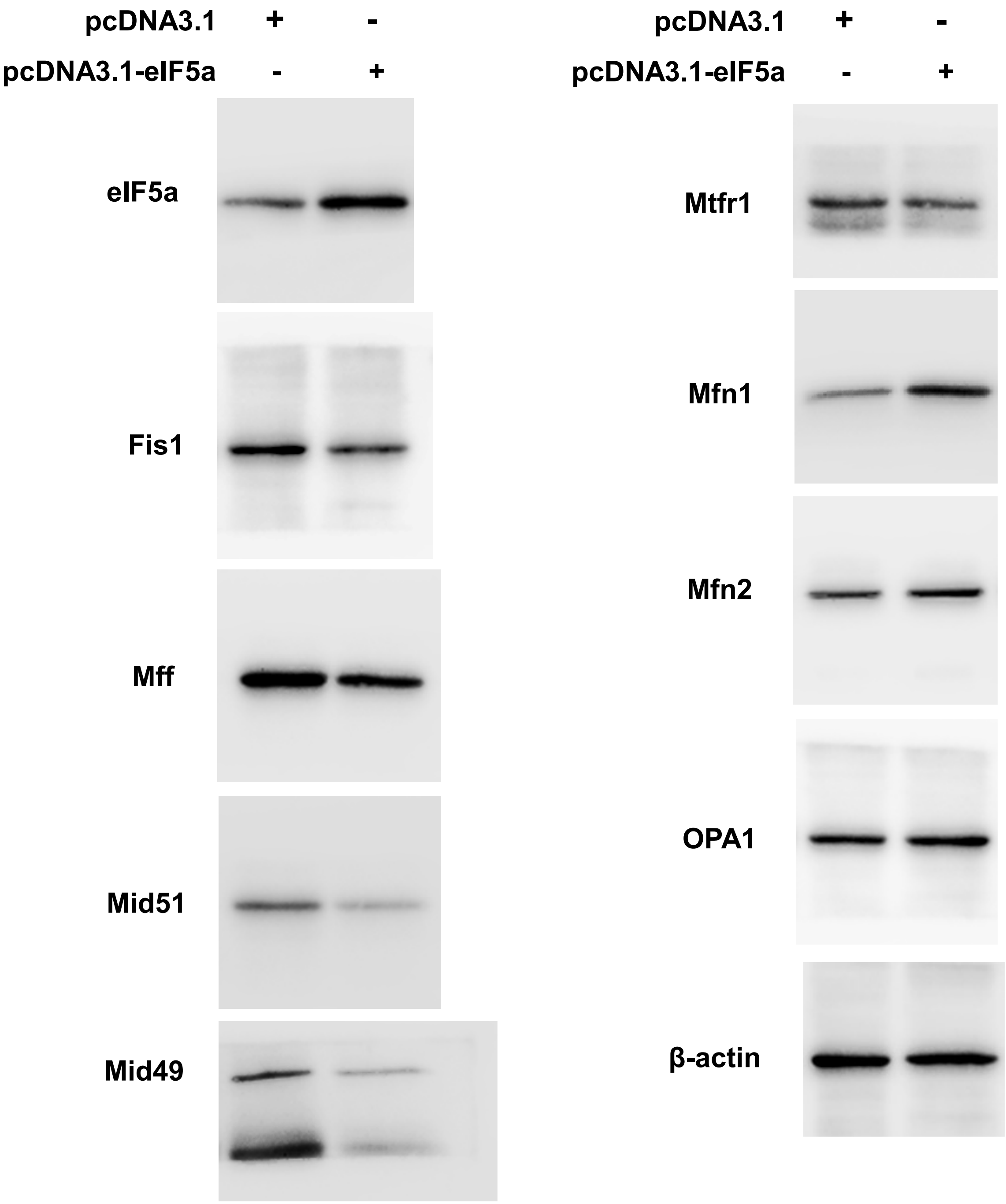

Full blot images-Figure S16A

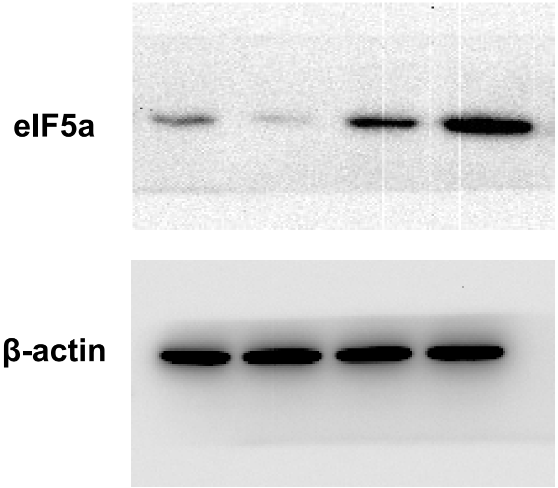

Full blot images-Figure S16B

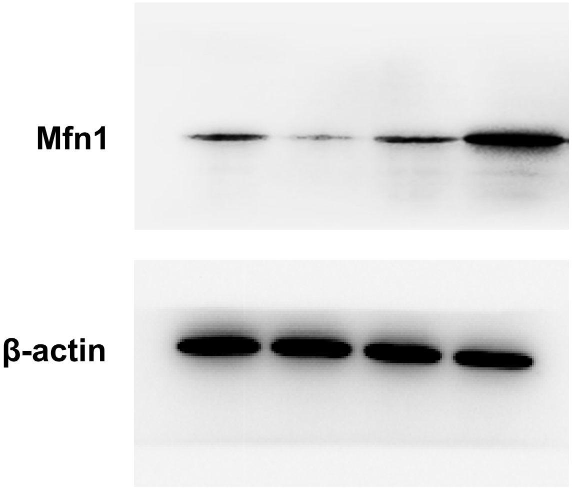

Full blot images-Figure S17E

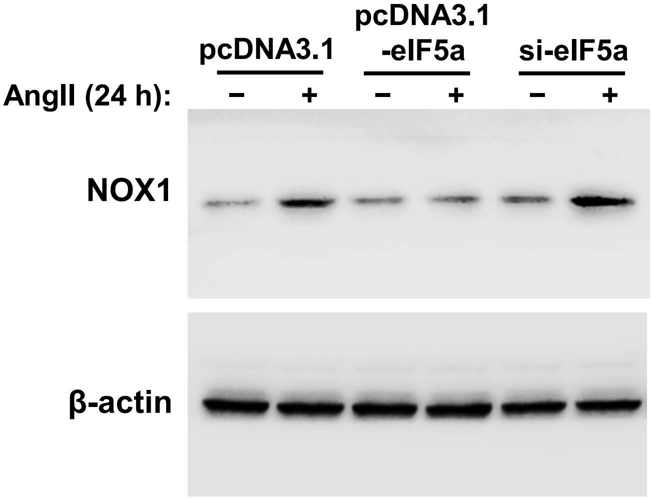

Full blot images-Figure S17F

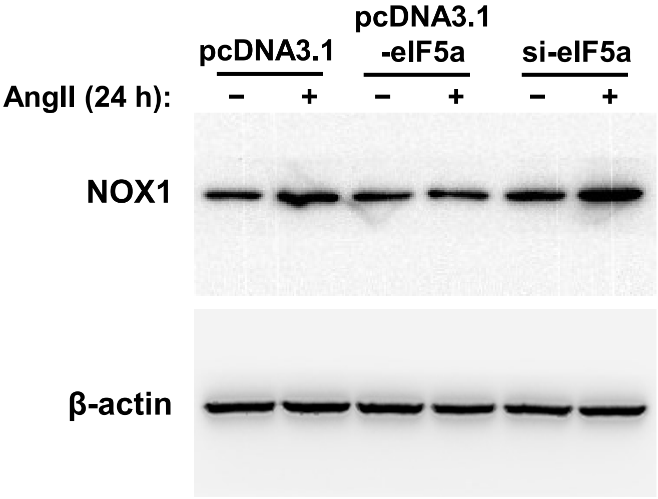

Supplement: S1 Blots — The file “S1_Blots.pdf” covers all uncropped western blot images, including size standards and descriptions. (PDF) [file pbio.3000808.s021.pdf]
