# Supplementary material for: Klf5 down-regulation induces vascular senescence through eIF5a depletion and mitochondrial fission
Source: PLoS Biol. 2020 Aug 20;18(8):e3000808. doi: 10.1371/journal.pbio.3000808 (PMC7462304; doi:10.1371/journal.pbio.3000808)
Supplement: Attachment — Submitted filename: Revised_Response_to_reviewers.docx [file pbio.3000808.s022.docx]

| Dear PhD Ines Alvarez-Garcia,  Thank you very much for giving us an opportunity to resubmit our manuscript entitled “eIF5a reduction elicited by Klf5 downregulation leads to cell senescence through inducing mitochondrial fission in vascular smooth muscle cells” (Manuscript No: PBIOLOGY-D-19-03711R1).  We appreciate your concerns (e.g. several experiments need to be performed to confirm some of the findings as well as to add several controls and clarifications), We have therefore performed a number of new experiments and extensively revised the manuscript  according to the reviewer’s comments.  In particular, Δψm and the activities of mitochondrial respiratory chain complexes I, II and IV were determined to evaluate mitochondrial functions, and the different roles of Klf5 in early and late stages of AAA were investigated. The role and underlying mechanism of excessive ROS generation and antioxidative treatment were explored. Several controls in experiments and clarifications in Figure Legends were added.  All changes have been highlighted in red color in the revised manuscript. Point by point responses to the reviewers’ comments are listed below this letter. |  |
| --- | --- |

Thank you and all the reviewers for the kind advice. It is our hope that this revised version of the manuscript meets the rigorous standards for the *Plos Biology*. Look forward to hearing from you.

Yours sincerely,

Jin-kun Wen

Professor of Biochemistry and Molecular Biology

Department of Biochemistry and Molecular Biology,

Hebei Medical University,

361 Zhongshan East Road, Shijiazhuang050017, China

Tel: 86-311-86265563

Fax: 86-311-86696180

E-mail: [wjk@hebmu.edu.cn](mailto:wjk@hebmu.edu.cn)

**Point-by-point response to referees**

**Rev. 1:**

This study examined the function of Krüppel-like factor 5 (Klf5) in abdominal aortic aneurysm (AAA). The authors found that Klf5 deficiency attenuates, while overexpression promotes mitochondria fusion. Mechanistically, the authors proposed that loss of Klf5 upregulates fission-related gene expression, which leads to enhanced ROS generation and vascular smooth muscle cell senescence. The findings are very interesting. However, more work needs to be done to reveal the role Klf5 plays in mitochondrial dynamics and function, as well as reveal its contribution to AAA, especially ruptured AAA.

1. Please divide the patients into "ruptured" and "unruptured" groups and report separately. Are these two groups comparable in terms of demographic and clinical characteristics?

A: Thanks for your careful review and good suggestion. We divided the patients into AAA and ruptured AAA groups in terms of demographic and clinical characteristics. As shown in Table 1, except for pain and increased aortic diameter and lipidemia, there is no meaningful difference between the two sets of data. This is probably associated with the fact that the number of samples in ruptured AAA group is too small.

2. Fig. 1h, there are Klf5 positive cells that are SMA negative, what are those cells?

A: Thanks for your suggestion. According to our previous study (supplemental information), Klf5-positive cells that are SMA negative mainly are macrophages that have infiltrated into the vascular wall as confirmed by immunofluorescence staining of F4/80, Klf5 and DAPI.

Reference is as follows: Ma D, Zheng B, Suzuki T, et al. Inhibition of KLF5-Myo9b-RhoA Pathway-Mediated Podosome Formation in Macrophages Ameliorates Abdominal Aortic Aneurysm. Circulation Research, 2017, 120(5):799-815.

3. What is the cause of death in the smcKlf5^−/−^ mice? Rupture of aortic aneurysm or cardiac problems? How is their cardiac function after Ang II treatment?

A: Thanks for your questions. In addition to the aneurysm rupture, we did find that the impaired cardiac function in all the mice infused chronically with Ang II. More importantly, Klf5 deletion led to worse cardiac function when echocardiography was used to assess cardiac function at 4 weeks post-infusion. As shown in S5 Fig, the left ventricular ejection fraction and shortening fraction were decreased in the smcKlf5^−/−^ mice and older mice relative to young WT mice (ejection fraction: 61.5 ± 6.6 % and 57.2 % vs. 72.1 ± 1.8 %, p < 0.05; shortening fraction: 44.7 ± 9.0 % and 43.5 % ± 9.7 % vs. 60.1 ± 1.0 %, p < 0.05), and the decreases were more obvious in older smcKlf5^−/−^ mice (ejection fraction: 45.9 ± 4.8 % vs. 72.1 ± 1.8 %, p < 0.01; shortening fraction: 37.6 ± 4.8 % vs. 60.1 ± 1.0 %, p < 0.01, respectively, S5A and 5B Fig). Accordingly, there was a significant increase in dimensions of left ventricle at systole and diastole in the smcKlf5^−/−^ mice and older mice relative to young WT mice (systole: 2.4 ± 0.5 and 3.2 ± 1.0 vs. 1.0 ± 0.1, p < 0.05, respectively; diastole: 3.5 ± 0.8 and 3.0 ± 0.5 vs. 2.5 ± 0.1 %, p < 0.05, respectively), and the increases were higher in older smcKlf5^−/−^ mice (systole: 3.2 ± 0.8 vs. 1.1 ± 0.08, p < 0.01; diastole: 4.1 ± 0.8 vs. 1.0 ± 0.1, p < 0.01, respectively, S5C and 5D Fig).

We have supplied the relevant descriptions (red color) in the part of Methods and Results.

In addition, it has been reported that the loss of Klf5 expression has led to a disorder of fatty acid metabolism and a decrease in ATP levels in the mouse cardiomyocytes. This phenomenon was also observed in mouse abdominal aortic tissues.

Reference is as follows: [Drosatos K](javascript:void(0);), [Pollak NM](javascript:void(0);), [Pol CJ](javascript:void(0);), et al. Cardiac Myocyte KLF5 Regulates Ppara Expression and Cardiac Function. Circulation Research, 2016, 118(2): 241–253.

4. The authors showed that one gene, elF5a, is the direct target of Klf5. How about the rest of the genes identified by RNAseq that were differentially expressed in Klf5 deficient or overexpressed cells? The expression of these genes were changed at the transcriptional level, if they were not regulated by Klf5, what is the direct regulator?

A: Thanks for your suggestion. We used western blot analysis to further validate the differentially expressed genes identified by RNA-seq and qRT-PCR in Klf5-deficient or Klf5-overexpressed mouse VSMCs. As shown in S9A and 9B Fig, the expression of Cox6a2 and Atp5b was downregulated or upregulated by Klf5 knockdown or overexpression, respectively, but the luciferase activity assay showed that these two genes were not regulated by Klf5 (S9C and 9D Fig), indicating that Klf5 regulation of Cox6a2 and Atp5b expression might be secondary or indirect.

In fact, we found that Klf5 knockdown or overexpression obviously affected the expression of Atp5b gene, which is closely associated with mitochondrial morphology. Unfortunately, we still have not elucidated the underlying link between Klf5 and Atp5b expression. It should be highlighted and emphasized in our further research. We hope reviewer will agree with our interpretation.

5. What is the cause of mitochondria fission when Klf5 is lost? Is it because that the fusion-related genes are repressed? Or is it because that mitochondrial function is impaired due to the downregulation of ATP production, and the changes in the expression of mitochondria dynamics-related genes are secondary?

A: Thank you for your suggestion. This is indeed a question worthy of consideration. Deregulation of mitochondrial dynamics, which is associated with the development of some cardiovascular diseases, can be attributed to altered expression of the mitochondrial fusion- and division-related genes (e.g. Mfn1/2 and Drp1). Recent reports show that Mfn1 is an important regulator of mitochondrial morphology, function and cell apoptosis. For example, Yang et al. reported that transfection of a miRNA-142a-5p mimic into skeletal muscle cells (C2C12) downregulated Mfn1 expression, leading to extensive mitochondrial fragmentation, depolarization of mitochondrial membrane potential (Δψm) and accumulation of ROS, accompanied by mitophagy activation and apoptosis. Importantly, these effects were attenuated by overexpression of Mfn1. Consistent with these reports, our results showed that chronic Ang II stimulation-induced Klf5 downregulation contributed to low expression of eIF5a in VSMCs, which in turn decreases the expression of Mfn1 and thus attenuates the interaction of eIF5a with Mfn1, subsequently leading to accumulation of ROS and excessive consumption of ATP. Therefore, impaired mitochondrial function might be caused by the aberrant expression of mitochondria dynamics-related genes.

We supplied the relevant descriptions in the discussion section in red word. Please check.

References are as follows:

Yang X, Xue P, Chen H, et al. Denervation drives skeletal muscle atrophy and induces mitochondrial dysfunction, mitophagy and apoptosis via miR-142a-5p/MFN1 axis. Theranostics, 2020;10(3): 1415-1432.

Wang T, Zhai M, Xu S, et al. NFATc3-dependent expression of miR-153-3p promotes mitochondrial fragmentation in cardiac hypertrophy by impairing mitofusin-1 expression. Theranostics, 2020;10(2): 553-566.

6. What is the physiological significance of the interaction between elF5a and Mfn1? The results suggested that overexpression of either one was sufficient to rescue the mitochondrial morphology, then is their interaction necessary?

A: It is really true as Reviewer suggested that single overexpression of elF5a or Mfn1 was sufficient to rescue the mitochondrial morphology, but co-expression of elF5a and Mfn1 had significantly more potent effect. Moreover, we found that eIF5a-enforced expression increased Mfn1 expression, whereas Mfn1-enforced expression did not affect eIF5a expression (S13A and 13B Fig). At the same time, eIF5a overexpression obviously increased its co-localization with Mfn1 and facilitated the formation of network-like mitochondria, whereas eIF5a knockdown had the opposite effects (Fig 6C, S12A Fig).These findings suggest that Mfn1 resides downstream of eIF5a, and that eIF5a can interact with Mfn1. Although single overexpression of elF5a or Mfn1 could partially rescue the mitochondrial morphology, eIF5a interaction with Mfn1 is necessary for the regulation of mitochondrial dynamics.

We supplied the relevant descriptions in the Results section in red word.

7. In the wild type cells after 5 days of Ang II treatment, the mitochondria in both wild type and Klf5-/- cells were fragmented, while after 1 day of treatment, the morphology of the mitochondria in wild type and KO were different. Moreover, Klf5 was recruited to elF5a promoter after 24 hr of Ang II (Fig. 5g and h). Therefore, the rescue experiment, which meant to demonstrate the effects of Klf5 in AngII stimulated mitochondrial fission, should also be done after 1 day of treatment, and see if enforced expression of Klf5, elF5a or Mfn1 could reverse the fission phenotype in the KO cells.

A: Thank you for your good suggestion. In the revised manuscript, we overexpressed Klf5, elF5a or Mfn1 by transfecting Klf5^−/−^ VSMCs with expressing plasmids encoding Klf5, elF5a or Mfn1, respectively, and then treated the cells with AngII for 24 h. Our results showed that enforced expression of Klf5, elF5a or Mfn1 in Klf5^−/−^ VSMCs greatly reduced AngII-stimulated mitochondrial fission compared with empty vector-transfected Klf5^−/−^ VSMCs (S14 Fig).

We supplied these descriptions (red color) in the part of result.

8. How does the enhanced mitochondrial fission in VSMCs contribute to the exacerbated AAA outcome/rupture?

A: It has been well known that the structure and function of the mitochondrial network are regulated by the dynamic processes of fusion and fission. Perturbations in mitochondrial dynamics can result in impaired mitochondrial function [Mitochondrial dynamics, mitophagy and cardiovascular disease. [J Physiol.](https://www.ncbi.nlm.nih.gov/pubmed/26537557) 2016 Feb 1;594(3):509-25], and declining mitochondrial function may lead to the production of reactive oxygen species [Reactive Oxygen Species and Mitochondrial Dynamics: The Yin and Yang of Mitochondrial Dysfunction and Cancer Progression. [Antioxidants (Basel).](https://www.ncbi.nlm.nih.gov/pubmed/?term=Reactive+Oxygen+Species+and+Mitochondrial+Dynamics%3A+The+Yin+and+Yang+of+Mitochondrial+Dysfunction+and+Cancer+Progression) 2018 Jan 16;7(1)] which could impact the inflammatory status and metabolism of a cell. Moreover, mitochondria-derived ROS can mediate redox signaling or, in excess, cause cell injury and even cell death [[Ageing: The yin and yang of mitochondrial dysfunction. [Nat Rev Mol Cell Biol.](https://www.ncbi.nlm.nih.gov/pubmed/27211483) 2016 May 23;17(6):331].](http://www.ncbi.nlm.nih.gov/pubmed/27211483) In this study, we found that eIF5a reduction induced by Klf5 downregulation in senescent VSMCs contributed to low expression of eIF5a, which in turn decreases Mfn1 expression and the interaction between eIF5a with Mfn1, subsequently leading to excessive accumulation of ROS and VSMC senescence and thus exacerbating AAA outcome/rupture.

We supplied the relevant descriptions in the Discussion section in red word.

**Minor:**

1. In file S1.TIF, are supplementary figure S2, while supplementary figure S1 are in file S2.TIF. The same happened to S3 and S4, S5 and S6.

A: We are very sorry for our careless. Correction has been made in the revised version of manuscript.

2. The mitochondrial function was not carefully evaluated, except for the measurement of ATP.

A: Thanks for your valuable suggestion. In the revised manuscript, we evaluated carefully the effect of Klf5 deficiency in VSMCs on mitochondrial functions, including Δψm and the activity of mitochondrial respiratory chain complexes. The results showed that the Δψm and activity of mitochondrial respiratory chain complexes were significantly reduced in Klf5-deficient VSMCs compared to the WT cells, whereas adenovirus-mediated overexpression of Klf5 in Klf5-deficient VSMCs largely rescued these reductions (S10A-10E Fig).

We added the relevant descriptions (red color) in the part of Methods and Results.

**Rev. 2:**

This is an interesting manuscript with extensive data that characterize the roles of Klf5 in unruptured and ruptured abdominal aortic aneurysm (AAA). They found that Klf5 is downregulated in human ruptured AAA compared to unruptured AAA. Furthermore, the authors also showed downregulation of Klf5 in mouse ruptured (42 days) compared to unruptured AAA (28 days) induced by Ang II in ApoE-/- mice. To further characterize the specific function of Klf5 in the pathogenesis of AAA, they generated mice lacking Klf5 in SMCs and showed a role of Klf5 deficiency in mouse AAA biogenesis induced by Ang II. Subsequently, the authors found that loss of Klf5 in VSMCs enhanced mitochondrial fission via upregulation of the pro-fission proteins Drp1, Fis1 and Mtfr1 and downregulation of the pro-fusion protein Mfn1. Finally, they found that mitochondrial fragmentation in VSMCs is a result from eIF5a reduction elicited by Klf5 downregulation…

The data presented in this manuscript provide new insight into how KlF5 affects the AAA biogenesis through regulating the expression of eIF5a, and in turn eIF5a regulate mitochondrial dynamics in VSMCs. However, experiments that support their conclusions need to be improved. Therefore, although promising, the manuscript does not seem suitable for publication in its present form.

The major points that must be addressed:

1. First of all, throughout the entire manuscript, the authors used mouse primary VSMCs and human VSMCs in different experiments, but they were not clearly indicated in each experiment. For example, the results in Figure 3 were obtained from human VSMCs? Figure 4C, D and F from human VSMCs? Figure 4H from mouse primary VSMCs? The same question is found in Figures 5-7. It is not clear why the authors wanted to use mouse primary VSMCs or human VSMCs in different experiments.

A: We are very sorry for our unclear representation. We have clarified the origin of VSMCs in different experiments and revised the corresponding Figure legends. According to the purpose of the particular experiment，mouse primary VSMCs or human VSMCs were chosen, namely, when we observed the effect of Klf5 deficiency in VSMCs on mitochondrial fission, mitoROS production, mitochondrial dynamics-related gene expression, and so on, mouse primary VSMCs were isolated from smcKlf5^−/−^ mice (mice lacking Klf5 specifically in SMCs) or Klf5^flox/flox^ mice (control, equivalent to wild-type) and were used. In addition to these experiments, we used human VSMCs (ScienCell, no. 6110) to conduct the experiments shown in Figure 3.

2. The description in the Figure Legends is unclear and should be re-written to make the figure easily understood. The results in Figure 3 were obtained from human VSMCs.

A: We are very sorry for our unclear representation and the misunderstanding caused. We have revised the description in all the parts of the Figure Legends to make the figure easily understood. The results in Figure 3 have been clearly indicated as human VSMCs.

3. In Figure 1A and 1B, the author analyzed the expression pattern of Klf5 protein by western blotting in patient tissues of unruptured AAA (22 cases), ruptured AAA (4 cases) and 8 control samples. However, they only showed the WB results in part of analyzed samples. It is very important to show levels of Klf5 protein in each sample including 8 controls, 22 unruptured AAA and 4 ruptured AAA. Additionally, in Figure 1C and 1D, immunohistochemistry of tissue sections that were collected from paraffin-embedded samples? and the sections came from the patient samples in Figure 1A should be indicated.

A: Thank you for your careful review and good suggestion. We provided the WB results of each sample including 8 controls, 22 unruptured AAA and 4 ruptured AAA, please see Fig. 1A. Tissue sections for mmunofluorescence staining were prepared from frozen samples of the patient with unruptured AAA and ruptured AAA, and these sections came from the patient samples indicated in Figure 1A.

We supplied the relevant description (red color) in the part of Figure Legends.

4. In Figure 1E and 1F, mock control mice infused with saline at 28 and 42 days should be added.

A: Thanks for your suggestion. In the revised manuscript, we added mock control mice infused with saline for 28 and 42 days (n=10 for each group) in Figure 1E and 1F.

5. In Figure 1H, how many mice at indicated time points were analyzed by immunohistochemistry should be indicated. Similar to those analyzed in human AAA as shown in Figure 1A, the authors should assess expression of Klf5 by western blotting in mouse ruptured AAA and unruptured AAA induced by Ang II in ApoE-/- mice.

A: To indicate the number of mice for each time point, we have changed the Figure 1H into scatter plots (Figure 1J in the revised manuscript). n=4 for each time point. In addition, we performed western blot assay to assess Klf5 expression in mouse unruptured AAA and ruptured AAA induced by Ang II in ApoE^−/−^ mice, n=5 for each group (Figure 1H).

We supplied the relevant description (red color) in the part of Results.

6. Given the importance of abnormal Klf5 expression in human AAA, it will be interesting to analyze the expression pattern by western blotting in mouse unruptured AAA and ruptured AAA induced by Ang II in ApoE-/- mice (at least 5 samples will be analyzed in each group).

A: Thank you for your suggestion. We detected Klf5 expression in mouse unruptured AAA and ruptured AAA induced by Ang II in ApoE^−/−^ mice as well as in the aortas from control mice infused with saline, n=5 for each group (Figure 1H).

We supplied the relevant description (red color) in the part of Results.

7. In Figure 2G, the immunohistochemistry of tissue sections (paraffin-embedded) from tumor areas of mouse unruptured AAA (at 3 month) and ruptured AAA (at 18 months) or should be clearly indicated. Additionally, the immunohistochemical images in Figure 2J are too low magnification to recognize the CD45-positive cells.

A: Thank you for your suggestion. In Figure 2G Legends of the revised manuscript, we clearly indicated that the tissue sections for DHE and mitoSOX staining were prepared from AAA samples of 3-month-old and 18-month-old WT or smcKlf5^−/−^ mice infused chronically with Ang II. To recognize the CD45-positive cells, the immunohistochemical images in Figure 2J have been locally magnified.

8. In Figure 3A-3C, the mock controls should be added in each indicated time points (for example, at 1, 3 and 5 days). In Figure 3B and 3I, the authors should analyze Ki-67-positive cells (%) but not Ki-67 relative fluorescence.

A: Thank you for your suggestion. In Figure 3A-3C of the revised manuscript, we added the mock controls at 0, 1, 3 and 5 days. In Figure 3B and 3I, Ki-67-positive cells (%) were quantified from three different fields.

We added the relevant description (red color) in the Figure 3 Legend.

9. In Figure 4, it is very confused to use mouse or human cells in different experiments. The authors should clearly indicate human or mouse cells used in different experiments. For examples, Figure 4A and 4B were probably performed in mouse materials, whereas Figure 4C, 4D and 4F probably in human VSMCs, and 4G and 4H used mouse VSMCs?

For the experiments in Figure 4E and 4F, to compare the effect of knockout and overexpression of Klf5 on the levels of the mitochondria-shaping proteins, the authors should perform these experiments in the same species (in mouse or in human VSMCs). Therefore, the authors should evaluate expression of those proteins indicated in Figure 4E using human VSMCs depleted of Klf5 by siRNA, and repeat experiments as shown in Figure 4H to see whether knockdown and overexpression of Klf5 results in an opposite effect on mitochondrial morphology in human VSMCs. In Figure 4D, ablation of Klf5 in mouse primary VSMCs (?) changes the expression levels of Fis1, Drp1, Mtfr1, Mfn1 and Pink1. How about the expression levels of these proteins if Klf5 re-introduced into Klf5-deficient cells?

A: We are very sorry for our unclear representation and the misunderstanding caused by mouse or human VSMCs in different experiments. To observe the effect of Klf5 deletion in VSMCs on mitochondrial dynamics-related gene expression and mitochondrial fission, mouse primary VSMCs were isolated from smcKlf5^−/−^ mice (mice lacking Klf5 specifically in SMCs) or Klf5^flox/flox^ mice (control, equivalent to wild-type) and were used in all the experiments shown in Figure 4. We have revised the description in the Figure 4 Legend. Moreover, we assessed the effects of overexpression and knockdown of Klf5 in human VSMCs on the expression of mitochondrial dynamics-related proteins and mitochondrial morphology (S8B and 8C Fig). Also, we infected Klf5-deficient VSMCs with pAd-Klf5 to assess the effect of Klf5 re-introduction on the expression of mitochondrial dynamics-related proteins. Western blotting showed that expression levels of Fis1, Drp1, Mtfr1, Mfn1 and Pink1 were restored in Klf5 re-introducting Klf5^−/−^ VSMCs (S8A Fig). These results further verified the importance of Klf5 in the regulation of mitochondrial dynamics.

We added the relevant descriptions (red color) in part of Results.

10. In Figure 5, were all the experiments performed in human cells? This should be indicated in each figure legend. The levels of Klf5 should be added in Figure 5D.

A: We are very sorry for our unclear representation. In Figure 5, all the experiments were performed in mouse VSMCs. We revised the Figure 5 legend to clarify the origin of VSMCs. Band intensities of eIF5a in Figure 5D were measured and normalized to β-actin and were shown at the upper side of representative Western blot (n = 3).

11. Again, in Figure 6, were all the experiments performed in mouse primary VSMCs or human VMSCs? This should be indicated in each figure legend. In co-immunoprecipitation assays shown in Figure 6A, the input data should be added. In Figure 6C, the author suggested that overexpression of eIF5a promotes mitochondrial fusion via an enhanced interaction with the pro-fusion protein Mfn1, whereas knockdown of eIF5a leads to an opposite effect, but they did not show the data about mitochondrial morphology analysis. Moreover, overexpression or knockdown of eIF5a seems to have an effect on levels of Mfn1 in cells as seen in Figure 6C. Thus, to better understand the potential role of eIF5a in regulating mitochondrial dynamics, the authors further evaluate the effects of overexpression or knockdown of eIF5a on the levels of Fis1, Mff, MIEF1/Mid51, MIEF2/Mid49, Drp1, Mtfr1, Mfn1, Mfn2 and OPA1 (by western blotting).

A: We are very sorry for our unclear representation. In Figure 6, all the experiments were performed in mouse VSMCs. We have revised the Figure 5 legend to clarify the origin of VSMCs.

In the revised manuscript, we have added the input data in Figure 6A. The data about mitochondrial morphology analysis in eIF5a-overexpressed or knocked down VSMCs are shown in S12A and 12B Fig. Additionally, according to your suggestion, we examined the effects of eIF5a overexpression on the levels of Fis1, Mff, MIEF1, MIEF2, Drp1, Mtfr1, Mfn1, Mfn2 and OPA1 by western blotting. As shown in S12C Fig, eIF5a overexpression significantly enhanced Mfn1, but attenuated Fis1 and Mff expression. We have supplied the relevant descriptions (red color) in part of Results.

12. In Figure 8A, the authors should use scatter plots that show all the individual data points and standard deviation (SD) to represent variation of eIF5a expression in human unruptured (n=22), ruptured (n=4) AAA and adjacent control arotas (n=8).

A: Thank you for your suggestion. In the revised manuscript, we have converted the column chart in Figure 8A to a scatter plots to show all the individual data points.

**Rev. 3:**

In this manuscript, Dong Ma et al. investigate the functional relationship of KLF5 with eIF5a and their involvement in the formation and rupture of abdominal aortic aneurysm via regulation of the proliferation of vascular smooth cells and induction of oxidative stress. This is an interesting study with potential ramifications in understanding of the molecular mechanisms that underlie biology of aneurysms, which may eventually lead to therapeutic interventions. The authors performed a combination of in vivo and in vitro experiments along with analyses of human samples, which signify further the translational potential of their findings. Here are the comments of this reviewer:

1. Although it is clear that KLF5 expression is upregulated at the early stage of the AAA and its expression returns back to normal levels at the late stage, it remains elusive how the authors came up with the conclusion that KLF5 is protective in late phase, when the disease has already reached the maladaptive stage. The biphasic pattern of KLF5 expression requires pharmacologic interventions at the early vs. late stage in order to identify the critical stage that KLF5 suppression may be beneficial or detrimental. The authors can apply pharmacologic treatment (ML264) in mice treated with AngII aiming to block KLF5 expression at the early stage only vs. continuous treatment (early + late stage) and monitor progression of the disease.

A: Thanks for your good suggestion. We pharmacologically suppressed the expression of Klf5 by injecting intraperitoneally ML264 (10 mg/kg, every two days) into mice infused with AngII for 14 days (early stage) and 42 days (early + late stage). Ultrasound imaging showed that ML264 significantly suppressed AngII-induced aortic dilation at the early stage compared to the vehicle control, whereas ML264 accelerated AngII-induced aortic dilation upon continuous administration by 42 days (early + late stage) (S2A and 2B Fig). Consistently, HE staining showed that ML264 treatment reduced aortic luminal dilation and wall thinning in Ang II-infused mice at the early stage, while accelerating luminal dilation, aortic dissection and wall thinning upon continuous administration by 42 days compared to the vehicle control (S2C Fig).

We have added the relevant descriptions (red color) in the part of Methods and Results.

2. In light of the need for investigation of the association of the biphasic alteration in KLF5 expression, the in vitro experiments of combined treatment with AngII and Ad-KLF5 or Ad-shKLF5 (Fig 3D, 3E) need to include more time points (Day 0, 1, 3, 5) as for Fig 3A-C and not only Day 0 and Day 5.

A: Thank you for your suggestion. We detected SA-β-gal-positive cells, ROS, Ki-67-positive cells and apoptosis cells after cells were treated with AngII and Ad-KLF5 or Ad-shKLF5 for 0, 1, 3, and 5 days, and added these results in Fig 3D-3H of the revised manuscript.

3. The authors need to demonstrate intensity of AngII signaling following long-term treatment of mice with the stimulator of AAA formation. Previous studies (e.g. Gao D.et al.; PLoS One. 2015) have shown that AT1 receptor antagonist (losartan) attenuates KLF5 expression. Thus, it is possible that the attenuation in KLF5 expression that is observed in the late stage of the disease is an effect of desensitization of the AngII receptor and not an active event of the AAA pathophysiology.

A: Thanks for your valuable suggestion. To examine intensity of AngII signaling following long-term treatment of mice with AngII, we measured the blood pressure of mouse AAA model by the tail-cuff method and performed Western blot to detect extracellular signal-regulated kinase 1/2 (ERK1/2) signaling, which is one of signal pathways activated by AngII binding to the AT1 receptor, and the expression of β-arrestin2 that mediates desensitization of G protein-coupled receptors (GPCRs) in AngII-induced mouse AAA model and in WT or Klf5^−/−^ VSMCs. As shown in S3A Fig, there was a sustaining high blood pressure in long-term treatment of mice with AngII. As expected, long-term treatment with AngII (28 and 42 days) significantly increased the expression level of phosphor-ERK1/2, and β-arrestin2 expression also had a modest increase (S3B Fig). Considering that our mRNA microarray analysis showing a 3.2-fold increase of β-arrestin2 expression in AngII-injured aortas from smcKlf5^−/−^ mice relative to normal abdominal aortas, and that Klf5 deficiency in VSMCs enhanced β-arrestin2 expression (S3C Fig), it is reasonable to conclude that KLF5 downregulation at the late stage of AAA could result in β-arrestin2 upregulation through an as yet unidentified mechanism. Based on the evidence that blood pressure of mice was elevated by long-term treatment with AngII, and intensity of AngII signaling was strengthened, it can be concluded that the attenuation in KLF5 expression at the late stage of the disease could not be an effect of desensitization of the AngII receptor. However, the complicated pathophysiology of AAA development and progression could be a main cause for Klf5 downregulation. We hope reviewer will agree with our interpretation.

We have added the relevant descriptions (red color) in the part of Methods and Results.

Reference is as follows:

Trivedi D B, et al. β-arrestin2 Deficiency Attenuates Abdominal Aortic Aneurysm Formation in Mice. Circulation Research, 2013, 112(9):1219-U64.

4. Oxidative stress is now presented in a way that is disconnected by the biochemical pathways that underlie it. The authors propose that Drp1-mediated fission is over-activated and leads to accumulation of fragmented mitochondria that account for oxidative stress. This presentation precludes from understanding the source of reactive oxygen species, accumulation of which is critical for the development of the AAA. The authors should investigate further the association of AngII signaling, KLF5, eIF5α with the expression of proteins that are involved in enzymatic and non-enzymatic sources of ROS.

A: Because it has been known that NOX1 and NOX4 are key enzymes responsible for ROS generation in VSMCs [Hu antigen R is required for NOX-1 but not NOX-4 regulation by inflammatory stimuli in vascular smooth muscle cells. Journal of Hypertension, 2016, 34(2):253-265], we determined the association of AngII signaling, Klf5 and eIF5α with NOX expression. To do this, mouse VSMCs were transfected with expression vectors for Klf5 and eIF5α or siRNA against Klf5 and eIF5α, respectively, and then treated with AngII for 0, 1, 3, 5 days, the expression of NOX1 and NOX4 was detected by RT-qPCR. As shown in S17A-17D Fig, AngII treatment increased NOX1 expression, peaked at 24 h after treatment with AngII and gradually declined thereafter. Overexpression of Klf5 or eIF5a significantly decreased, whereas their knockdown increased, NOX1 expression. However, single treatment with AngII did not significantly affect NOX4 expression. We further confirmed these observations at the protein level by western blot (S17E and S17F Fig). These findings suggest that AngII promotes ROS production at least in part via upregulating NOX1 expression, whereas Klf5 or eIF5a decreases AngII-induced ROS formation through suppressing NOX1 expression.

We have supplied the relevant descriptions (red color) in the part of Methods and Results.

5. Mitochondrial function assays e.g. activity of mitochondrial enzymes, Seahorse analysis, along with assessment of markers for mitochondrial number (mtDNA: nuDNA) and replication (mtTFA, PGC1 etc.) in the early and late stage of AAA development in mice and/or in vitro would help to identify whether impairment of mitochondrial health is a cause or consequence of oxidative stress and eventually a major driver for the development of the disease.

A: Thank you for your good suggestion. To better evaluate the effect of Klf5 deficiency in VSMCs on mitochondrial functions, we detected the mitochondrial membrane potential (Δψm) and the activity of mitochondrial respiratory chain complexes in WT and Klf5^−/−^ VSMCs as well as in Klf5 re-introducting Klf5^−/−^ VSMCs. As shown in S10A-10E Fig, the Δψm and the activity of mitochondrial respiratory chain complexes I, II and IV were significantly reduced in Klf5-deficient VSMCs compared to the WT cells, whereas adenovirus-mediated overexpression of Klf5 in Klf5-deficient VSMCs rescued them to large extend.

In addition, to identify whether impairment of mitochondrial functions is a cause or consequence of oxidative stress and eventually a major driver for AAA development, we detected the mitochondrial functions of VSMCs treated with AngII for different times and assessed the markers for mitochondrial number (mtDNA) and replication (mtTFA, PGC1α). As shown in S10F-10I Fig, AngII treatment attenuated mitochondrial complex I, II and IV activities and ATP levels in a time-dependent manner. Correspondingly, AngII also decreased time-dependently the protein expression of PGC-1α and mtTFA as well as the number of mtDNA. We speculated that reciprocal causation between mitochondrial function impairment and oxidative stress co-operatively drives AAA development and progression.

We have supplied the relevant descriptions (red color) in the part of Methods and Results.

6. Treatment of mice with an anti-oxidant in the early vs. late stage of AAA would help to identify the extent of the involvement of oxidative stress in the effect of KLF5 and eIF5α inhibition in promoting the disease.

# A: Thank you for very helpful and valuable comments. In this study, we demonstrated that Klf5-dependent regulation of eIF5a/Mfn1 is required for mitochondrial dynamics and homeostasis processes, and that eIF5a reduction elicited by Klf5 downregulation contributes to mitochondrial fission, which in turn leads to excessive ROS generation and thus enhances VSMC senescence. These findings imply that treatment of AngII-induced mouse AAA models with ROS scavenger with high anti-oxidative capacity would be helpful for determining the extent of the involvement of oxidative stress in AAA development. Therefore, mouse VSMCs were pre-treated with N-acetyl-L-cysteine (NAC, an ROS scavenger), followed by treatment with AngII for 1 or 5 days. The results showed that NAC pre-treatment significantly attenuated mtROS levels in Ang II-stimulated VSMCs for 1 or 5 days (S18A Fig). Moreover, pre-treatment with NAC significantly reduced AngII-induced mitochondrial fission in AngII-treated VSMCs for 1 day relative to those untreated with NAC, but NAC had little inhibitory effect on mitochondrial fission in AngII-treated cells for 5 days (S18B Fig). These results suggest that antioxidative treatment with the ROS scavenger may partially eliminate excessive ROS in the early stage of AAA, preventing ROS-induced mitochondrial fission.

We have supplied the relevant descriptions (red color) in the part of Methods and Results.

7. What is the level of expression of Mfn1 in Fig. 6F and of eIF5α in Fig. 6G?

A: According to your suggestion, we measured the fluorescence intensity of Mfn1 in Fig. 6F and eIF5α in Fig. 6G, and quantification of the relative fluorescence intensity of these two proteins are shown in S13A and 13B Fig.

8. What is the effect of combined AngII treatment and KLF5 or eIF5a overexpression in ATP content of VSMCs?

A: Thanks for your suggestion. In the revised manuscript, mouse VSMCs were infected or transfected with Ad-Klf5 or pcDNA3.1-eIF5a and then treated with AngII for 5 days. ATP content was detected by ATP bioluminescent assay. As shown in S15 Fig, overexpression of Klf5 or eIF5a combined with AngII treatment partly rescued the ATP falling induced by AngII treatment for 5 days.

We have supplied the relevant descriptions (red color) in the part of Results.

9. Minor: Rearrange panels of Fig 6, so that they are in order.

A: Thanks for your careful checks. We are sorry for our carelessness. In the revised manuscript, we rearranged panels of Figure 6.
