# Supplementary material for: Klf5 down-regulation induces vascular senescence through eIF5a depletion and mitochondrial fission
Source: PLoS Biol. 2020 Aug 20;18(8):e3000808. doi: 10.1371/journal.pbio.3000808 (PMC7462304; doi:10.1371/journal.pbio.3000808)
Supplement: Attachment — Submitted filename: 2nd Response to Reviewers comments.docx [file pbio.3000808.s023.docx]

Dear Senior Editor Ines Alvarez-Garcia,

Thanks very much for your kind work and consideration on publication of our paper entitled “Klf5 downregulation induces vascular senescence through eIF5a depletion and mitochondrial fission” (Manuscript No: PBIOLOGY-D-19-03711R2).

Thank you very much for your help in modifying the title of the paper. According to your suggestion, we have revised the title as followings: "Klf5 downregulation induces vascular senescence through eIF5a depletion and mitochondrial fission."

We appreciate reviewers very much for their constructive comments and suggestions. These comments are all valuable and very helpful for revising and improving our paper. We have carefully revised the manuscript according to the reviewers’ suggestions.

All changes have been highlighted in red color in the revised manuscript. Point by point responses to the reviewers’ comments are listed below this letter.

Thank you and all the reviewers for the kind advice again. It is our hope that this revised version of the manuscript meets the rigorous standards for the Plos Biology. Look forward to hearing from you.

Yours sincerely,

Jin-kun Wen

Professor of Biochemistry and Molecular Biology,

Department of Biochemistry and Molecular Biology,

Hebei Medical University,

361 Zhongshan East Road, Shijiazhuang050017, China

Tel: 86-311-86265563

Fax: 86-311-86696180

E-mail: wjk@hebmu.edu.cn

**Point-by-point response to referees**

**Rev. 1:**
The authors have addressed my questions with new experiments and data. I have a couple of minor points:

1. Supplementary Fig. 10H and I, although these results demonstrated that AngII treatment caused mitochondria dysfunction in a time dependent manner and suggested a causal relationship between AngII and the impairment of mitochondrial function, the role of Klf5 cannot be inferred from these data. Therefore, the conclusion should be revised.
**A:** Thanks for your careful comments and helpful suggestions. According to your suggestion, we deleted the conclusion regarding “Klf5 deficiency in VSMCs leads to mitochondrial fission and impairment of mitochondrial function” at the end of this paragraph.

2. Supplementary figure 10E, the label of the 3rd column should be "smcKlf5-/-+Ad-Klf5".
**A:** We are sorry for our carelessness. It has been corrected in the revised manuscript.

**Rev. 2:**
In the revised manuscript, the authors have addressed my concerns and I have no additional comments.
**A:** Thank you very much for taking your time to review our manuscript.

**Rev. 3:**
The authors have made a significant effort to address the comments of this reviewer.

The new experiments that they performed do not support the notion that KLF5 suppression at the late stage of AAA is a protective response (Fig S2A,B). In fact, this figure should be part of the main Figures panel and not in the Supplement. Accordingly, the final version should focus primarily on the detrimental effect of KLF5 in driving AAA following activation of the protein. Statements implying that KLF5 suppression at the end stage are protective need to be removed from the text because they do not seem to reflect on the actual biological events.
**A:** Thanks for your careful comments and constructive suggestions. According to your suggestion, Fig S2A, B in the Supplement have been moved to Figure 1 (main Figures) as panels K and L. At the same time, we removed statements “KLF5 suppression at the late stage are protective”, from the text.

The limited improvement in cells treated with an anti-oxidant (in vitro; Fig S18) and the lack of in vivo proof that suppression of oxidative stress per se can be protective limits the possibility that this is a driving force for the observed phenotype. The authors should add a "Limitations" paragraph at the end of the Discussion section stating that treatment of AAA with anti-oxidants can be only supplemental to other interventions that should target more essential pathways for the development of the disease.

**A:** Thank you for very helpful comments. According to the comment, we have added a "Limitations" paragraph at the end of the Discussion section as follows: A major limitation of the present study is that we did not study the protective effect of the anti-oxidant N-Acetyl-L-cysteine (NAC) on AngII-induced vascular injury. Moreover, in the cultured VSMCs, suppression of AngII-induced oxidative stress by NAC only partially improved mitochondrial morphology. In addition, AngII-induced production of inflammatory factors also plays an important role in the impairment of mitochondrial function and vascular senescence. Certainly, multiple mechanisms, such as oxidative stress, inflammatory response, and dysregulated mitochondrial dynamics, are involved in vascular senescence and AAA development and progression. Therefore, treatment of vascular senescence and AAA with anti-oxidants can be only supplemental to other interventions that should target more essential pathways for the development of the disease.
